# Multi-dimensional relationships among dementia, depression and prescribed drugs in England and Wales hospitals

**DOI:** 10.1186/s12911-022-01892-9

**Published:** 2022-10-07

**Authors:** Alok Joshi, Stephen Todd, David P. Finn, Paula L. McClean, KongFatt Wong-Lin

**Affiliations:** 1 Intelligent Systems Research Centre, Ulster University, Magee Campus, Derry~Londonderry, Northern Ireland, UK; 2 Department of Computer Science, University of Bath, Bath, UK; 3 Altnagelvin Area Hospital, Western Health and Social Care Trust, Derry~Londonderry, Northern Ireland, UK; 4 Pharmacology and Therapeutics, School of Medicine, Galway Neuroscience Centre, National University of Ireland Galway, Galway, Ireland; 5 Northern Ireland Centre for Stratified Medicine, Biomedical Sciences Research Institute, Ulster University, Magee Campus, Derry~Londonderry, Northern Ireland, UK

**Keywords:** Dementia, Alzheimer’s disease, Vascular dementia, Depression, Antidepressant, Antipsychotic, Antianxiety, Drug prescription, Hospital admission, Stay and discharge

## Abstract

**Background:**

Dementia is a group of symptoms that largely affects older people. The majority of patients face behavioural and psychological symptoms (BPSD) during the course of their illness. Alzheimer’s disease (AD) and vascular dementia (VaD) are two of the most prevalent types of dementia. Available medications provide symptomatic benefits and provide relief from BPSD and associated health issues. However, it is unclear how specific dementia, antidepressant, antipsychotic, antianxiety, and mood stabiliser drugs, used in the treatment of depression and dementia subtypes are prescribed in hospital admission, during hospital stay, and at the time of discharge. To address this, we apply multi-dimensional data analytical approaches to understand drug prescribing practices within hospitals in England and Wales.

**Methods:**

We made use of the UK National Audit of Dementia (NAD) dataset and pre-processed the dataset. We evaluated the pairwise Pearson correlation of the dataset and selected key data features which are highly correlated with dementia subtypes. After that, we selected drug prescribing behaviours (e.g. specific medications at the time of admission, during the hospital stay, and upon discharge), drugs and disorders. Then to shed light on the relations across multiple features or dimensions, we carried out multiple regression analyses, considering the number of dementia, antidepressant, antipsychotic, antianxiety, mood stabiliser, and antiepileptic/anticonvulsant drug prescriptions as dependent variables, and the prescription of other drugs, number of patients with dementia subtypes (AD/VaD), and depression as independent variables.

**Results:**

In terms of antidepressant drugs prescribed in hospital admission, during stay and discharge, the number of sertraline and venlafaxine prescriptions were associated with the number of VaD patients whilst the number of mirtazapine prescriptions was associated with frontotemporal dementia patients. During admission, the number of lamotrigine prescriptions was associated with frontotemporal dementia patients, and with the number of valproate and dosulepin prescriptions. During discharge, the number of mirtazapine prescriptions was associated with the number of donepezil prescriptions in conjunction with frontotemporal dementia patients. Finally, the number of prescriptions of donepezil/memantine at admission, during hospital stay and at discharge exhibited positive association with AD patients.

**Conclusion:**

Our analyses reveal a complex, multifaceted set of interactions among prescribed drug types, dementia subtypes, and depression.

**Supplementary Information:**

The online version contains supplementary material available at 10.1186/s12911-022-01892-9.

## Background

Dementia is considered as an age-related syndrome. Alzheimer’s disease (AD) is the most prevalent type of dementia that impairs cognitive abilities and interferes with an individual’s day-to-day life [1, 2]. Besides cognitive impairment, 90% of dementia patients also experience behavioural and psychological symptoms (BPSD), widely known as neuropsychiatric symptoms, at some stage of their illness. Typically, BPSD comprises symptoms such as anxiety, aggression, agitation, hallucinations, delusions, irritability, poor appetite, and abnormal sleep and motor behaviour [3]. Additionally, patients living with dementia often have other comorbidities which, at times are undiagnosed and difficult to manage [4]. In particular, dementia patients often live with multiple health conditions including psychosis which can arise from underlying psychiatric disorders (e.g. depression, schizophrenia, and bipolar disorders) or respiratory, urinary, cardiovascular and gastrointestinal conditions [5–10]. Thus, many patients, family members and caregivers require increased medical services that results in considerable healthcare costs [11].

There is currently no known cure for dementia, though available dementia treatment strategies aim to either alleviate certain symptoms or offer some relief of cognitive dysfunction associated with AD [12]. Acetylcholinesterase inhibitors (AChEI) (e.g. donepezil, rivastigmine and galantamine) belong to one such group of drugs [13, 14]. These drugs are the first-line therapy for mild to moderate AD, and work by increasing the brain’s acetylcholine level, which is known to be impaired in dementia [12, 15]. Memantine, a N-methyl-D-aspartate (NMDA) receptor antagonist that reduces glutamate signalling, is indicated in the treatment of moderate to severe AD [16].

Antidepressants, along with a range of mood stabilisers (lithium, anticonvulsant and antipsychotic medicines), are commonly used to treat psychiatric conditions [17–19]. Additionally, anticholinergic drugs are used to address a range of other conditions which are common in dementia (e.g. overactive bladder) [20]. However, particular care should be given while prescribing these drugs, as anticholinergic and sedative drugs are linked to cognitive dysfunction and higher mortality rates [21], especially in older individuals [22]. Additionally, there is evidence that long-term usage of some drugs (e.g. tolterodine, used in the treatment of overactive bladder) can increase the risk of dementia [23]. Selective serotonin reuptake inhibitors (SSRIs) belong to another class of drugs used in treating depression, including in elderly patients. However, there are differences in opinion on whether SSRIs are safe for dementia patients. For instance, some believe that these drugs (e.g. fluoxetine) provide neuroprotective effects and help in improving cognitive function [24]. Indeed, serotonin receptor targeted drugs have been suggested for the treatment of AD [25]. However, other studies suggest that long-term usage of SSRIs increases the risk of dementia [26].

Until now, various studies have been conducted to understand the associations among dementia, age, ethnicity, dementia, antipsychotic, and antidepressant medications but they are mainly limited to descriptive analyses with very few of them exploring their combinations [27]. For example, a regression study by Barnes and colleagues showed that patients with age of 70 or less, patient’s care settings (e.g. Private continuing care, residential home, nursing home), dementia subtypes (e.g. vascular dementia (VaD), AD, frontotemporal dementia (FtD)), and severity of the disease are closely associated with antipsychotic medications [28].

Generally, antipsychotic medications are known to show modest efficacy in the treatment of dementia patients who experience psychotic symptoms [29]. However, usage of antipsychotics is associated with numerous harmful side effects such as pneumonia, stroke, somnolence, urinary tract infection and extrapyramidal symptoms, with increased mortality risk [30]. Despite the knowledge of recognised harms of prescribing antipsychotics with limited benefits, clinicians often continue their previous behaviours and write these prescriptions, as non-pharmacological interventions are harder (more time-consuming, staff intensive, etc.). Indeed, there are expected associations therefore between what is already prescribed by the clinician at admission and what is added during admission or on discharge. Thus, the UK’s National Institute for Health and Care Excellence (NICE) guidelines state that individuals with dementia should only be prescribed antipsychotics when they cannot cope with psychotic symptoms and are at significant risk of harming themselves or others [12, 28]. Following the earlier NICE guidelines, a longitudinal retrospective cohort study by Donegan et al. [31] showed that in a ten-year period, prescription of dementia drugs had doubled while the prescription of antipsychotics was reduced significantly in patients diagnosed with dementia.

Overall, these studies although valuable, generally provide descriptive statistical analyses of specific features (e.g. age, ethnicity, dementia subtypes, or antipsychotic medications), and are limited in providing more holistic, multi-dimensional insights or relationships among specific antipsychotic, antidepressant, dementia drugs, dementia subtypes and neuropsychiatric disorders. Moreover, there is a lack of such investigation within the context of hospital admission, stay and discharge.

In this work, we address this by applying multi-dimensional data analytical methods to provide insights into drug prescribing practices. We will particularly focus on the association among antidepressant, antipsychotic, antianxiety, antiepileptic/anticonvulsant and dementia drugs with dementia subtypes and neuropsychiatric disorders (particularly depression), given their potential co-prescriptions and interactions. The study will focus on hospitals in England and Wales.

## Methods

### NAD data

**Fig. 1:**
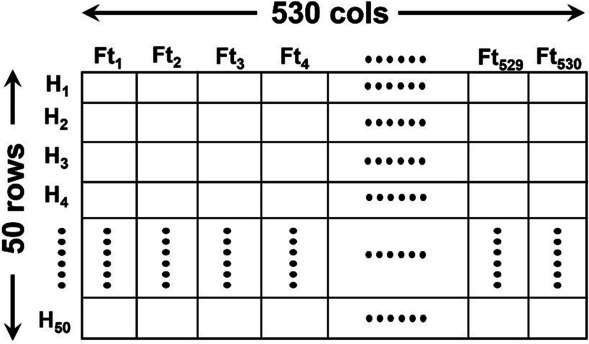
NAD dataset: The dataset consisted of 530 features (denoted by columns Ft_1_ to Ft_530_) and 50 hospitals across England and Wales (denoted by rows H_1_ to H_50_) (see Additional files 3, 6 for details of each feature). Note the “wideness” of the dataset

In this work, we made use of the National Audit of Dementia (NAD) dataset [32]. The NAD data describes psychotropic medication prescribed for the treatment of BPSD in patients admitted, between Feb and April 2019, to 50 (anonymised) hospitals in England and Wales. This data has about 530 features that are related to age, gender, ethnicity, first language, speciality team with whom patients spent the longest time (e.g., general medical, cardiac, cancer), primary diagnosis, delirium as a part of admitting condition, number of patients with dementia subtypes (AD, VaD, and FtD), with psychiatric diagnosis (most patients indicated depression or delirium during hospital admission), who died in hospital, or details about patients who discharged from hospital (Fig. 1). Additionally, the data contains details associated with patient’s later life care, length of stay in the hospital, place of residence before admission, place after discharge, and prescribed drugs (on admission, in hospital and on discharge).

Further, the dataset includes details of the total number of prescriptions, and number of antidepressants, antipsychotics, mood stabilisers, anxiolytics, anticonvulsant, and dementia-related drug prescriptions on admission, whilst in hospitals, and on discharge (see Additional files 3, 6 for further details). Hence, this is considered a “wide” dataset (Fig. 1), and dimensional reduction of the data is needed.

For each hospital, each feature is described in terms of numerical discrete values; these values are either zeros or discretized. We will focus our analysis only on AD, VaD, and FtD patients, given the lack of data for other dementia subtypes. In particular, within the context of hospital admission, stay and discharge, our work aims to elucidate the relationships among the number of patients with dementia, the number of patients with psychiatric disorders, and the number of prescriptions of antidepressants, antipsychotics, antianxiety, anticonvulsant drugs, mood stabilisers, and dementia treatment drugs.

### Data pre-processing and feature reduction

**Fig. 2:**
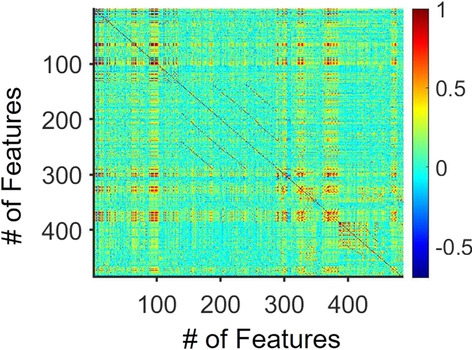
Correlation matrix for any pair of data features within 486 features. Colour bar: pairwise Pearson correlation values. These features (Ft) are: Ft1: total number of patient participated in the audit; Ft2 to Ft9: age related features; Ft10 to Ft14: gender specific features; Ft15 to Ft21: Ethnicity features; Ft22 to Ft25: language features; Ft26 to Ft34: patients with specific ward/team; Ft35 to Ft60: patients with primary diagnosis; Ft61 to Ft66: patients with delirium as a part of admitting condition; Ft67 to Ft77: patients with recorded dementia subtypes; Ft78 to Ft88: patients with psychiatric diagnosis; Ft89 to Ft91: patients died in the hospitals; Ft92 to Ft97: patients details related to discharge from the hospital; Ft98 to Ft99: patients receiving end of life care or care plan; Ft100 to Ft113: patients with length of stay recorded; Ft114 to Ft125: place of residence recorded before admission; Ft126 to Ft137: place of residence recorded after discharge; Ft138 to Ft187: total number of prescriptions of specific drugs at the time of admission; Ft188 to Ft239: total number of prescriptions of specific drugs in the hospital; Ft240 to Ft289: total number of prescriptions of specific drugs at the time of discharge; Ft290 to Ft298: number of prescriptions under different scenarios; Ft299 to Ft324: total number of prescriptions related to antipsychotic, hypnotics, antidepressant, dementia and anticonvulsants, same resumed/stopped at different time; Ft325 to Ft359: total number of new prescriptions related to antipsychotic, hypnotics, antidepressant, dementia and anticonvulsants during different time in hospital; Ft360 to Ft367: regular prescriptions related to antipsychotic, hypnotics, antidepressant, dementia and anticonvulsants; Ft368 to Ft385: total number of prescriptions related to antipsychotic, hypnotics, antidepressant, dementia and anticonvulsants; Ft386 to Ft421: prescriptions when reasons for prescription are recorded; Ft422 to Ft447: New prescription target symptoms recorded; Ft448 to Ft466: new prescriptions of antipsychotic, hypnotics, antidepressant, dementia and anticonvulsants are recorded by person/team; Ft467 to Ft486: prescriptions recommended for review or reviewed at different times.

**Fig. 3:**
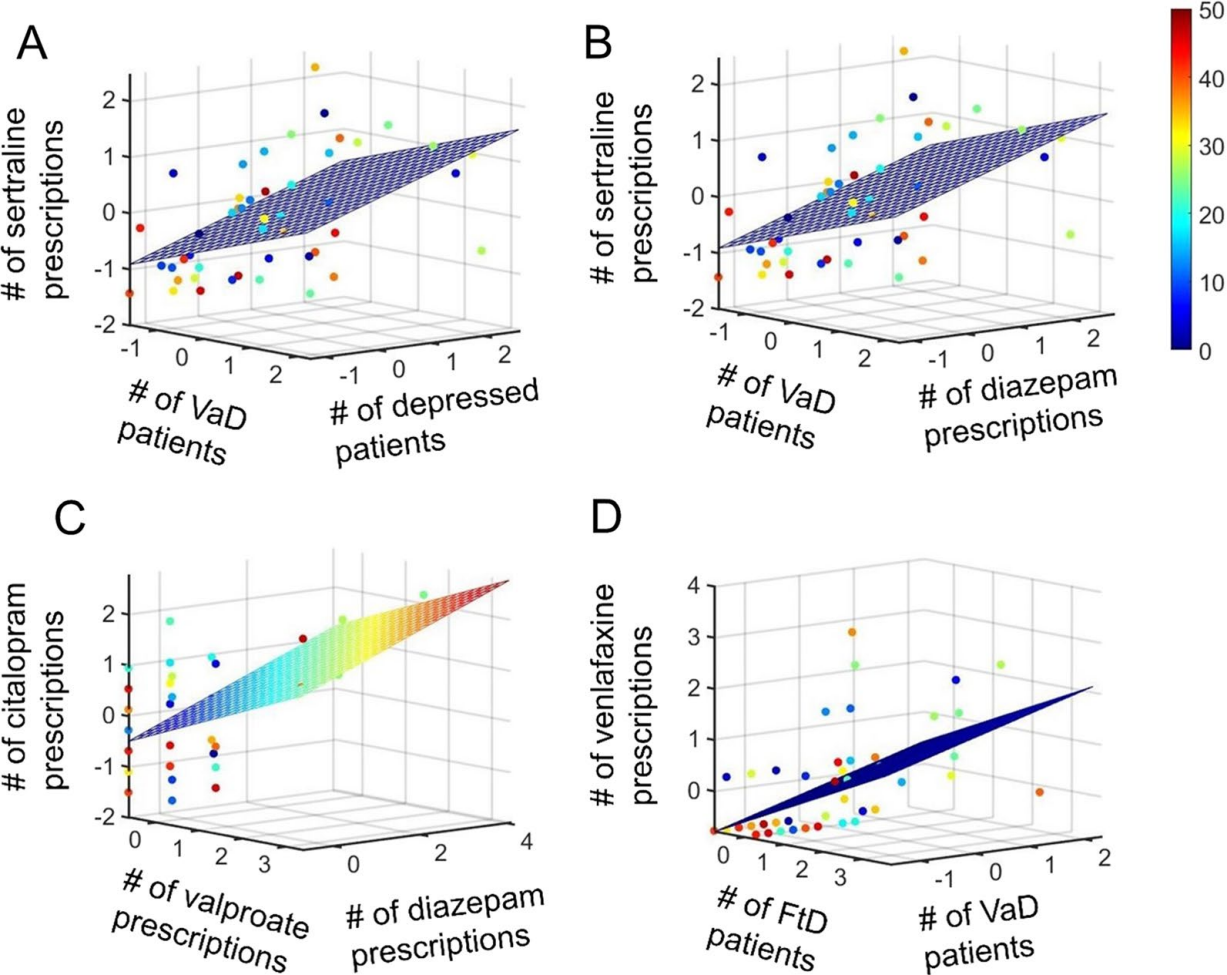
Associations among number of AD, VaD, FtD, and patients with depression, and prescribed drugs: **A** Relationship among number of sertraline prescriptions, VaD and depressed patients; **B** Number of sertraline prescriptions, number of VaD patients, and diazepam prescriptions; **C** Number of citalopram prescriptions, valproate, and diazepam prescriptions; **D** Number of venlafaxine prescriptions, number of FtD, and VaD patients, all are for hospital admission. Colour bar denotes data from the 50 different hospitals.

First, we pre-processed the dataset and removed those features that were assigned with zero values for all the hospitals. This reduced the number of features from 530 to 486. Following that, we selected 289 features that were related to the patient’s age, ethnicity, longest stay in hospital, and medications at the time of admission, during hospital and discharge (see Fig. 2 caption for details), and manually omitted the remaining features which were related to status (prescription continued or stopped), type (same or new prescription), time (during admission, hospitalisation or discharge) and reasons for prescriptions recorded, prescribed by person/team, reviewed at different times, as these were outside the scope of the study (for details see Ft290-Ft486, Fig. 3 caption, also see labels in dataset file: spotlight-data.csv). Additionally, we ignored features corresponding to the prescriptions where target symptoms were recorded during admission or while in hospital or at the time of discharge, and review of prescriptions to be held during the discharge, as these features are not drug-specific (e.g. related to antidepressant or dementia drugs) (see Additional file 5 for the list of selected features).

After that, we standardised the dataset and applied a prescribed Pearson pairwise correlation coefficient threshold with absolute value of above 0.4 to identify the more significant relationships between features [33]. Then, we selected the features (e.g. age, ethnicity, disorders, dementia subtypes, number of antidepressants, antipsychotics, mood stabilisers, anxiolytics, anticonvulsants, and dementia-related drug prescriptions) which are highly correlated with dementia subtypes (AD, VaD, and FtD). After that, we focused on these specific drug prescriptions and explored how they are correlated with the other features at various stages in the hospital (e.g. admission, stay, and discharge).

### Multiple regression analysis

After data pre-processing and reduction, we standardised all the features by calculating the z-score for each value and performed multiple regression analysis [34] on specific dementia or antidepressant drugs as a dependent variable and the other drugs (e.g. antidepressants, antipsychotics, antianxiety, anticonvulsant drugs, mood stabilisers, and dementia treatment drugs) and neurological/neuropsychiatric disorders (AD, VaD, FtD, and depression) as independent variables. This allowed a more holistic estimation of the value of our dependent variable based on the value of other multiple independent variables. We initially considered all the independent variables and evaluated their associated *p* values so that the estimated value calculated via a regression function was close to the known values. In cases where the *p* values were greater than 0.05, we ignored those independent variables and repeated the process with the rest of the variables until all the *p* values were less than 0.05, which we considered to be statistically significant. In this process, we removed the variable which had the highest *p* values, and repeated this step until all remaining variables had a *p* values of less than 0.05. However, we also noticed some other combinations of independent variables also contain *p* values within the statistically significant regime, we also considered those subsets as associated features.

We computed the correlation coefficients using MATLAB [35] and performed regression analysis using Bioinfokit package written in Python 3 [36].

## Results

### Correlation between sertraline and citalopram, and VaD, mirtazapine, venlafaxine, diazepam and valproate

The openly available National Audit of Dementia (NAD) dataset was used in our analysis. Prior to any analysis, we first pre-processed by removing data features (variables) that were not present for all the hospitals. This reduced the number of data features from 530 to 486. Then, to elucidate the association between any two of the selected features, we computed their pairwise Pearson correlation coefficient [37]. The resultant correlation matrix for these features is summarised in Fig. 2. In Fig. 2, we can also see that despite the large number of features in the data, only a subset had strong pairwise relationships (redder coloured regions). Then, we selected the prescription of drug-related features (number of prescriptions of antidepressants, antipsychotics, antianxiety, anticonvulsant, mood stabilisers, and dementia drugs) one at a time and compared the correlation coefficients using a coefficient threshold with absolute value above 0.4 (Methods).

In terms of antidepressant drugs, we considered the number of sertraline and citalopram drug prescriptions (Fig. 2, features Ft167, Ft219, Ft269 and Ft157, Ft208, Ft259 at hospital admission, during hospital stay, and discharge respectively). For their correlations with other drugs (antidepressants, mood stabilisers, antipsychotics, antianxiety, anticonvulsant, and anti-dementia) and disorders (depression and dementia subtypes), we first considered the number of sertraline prescriptions at the time of admission, acting as the dependent feature, and observed that this was positively correlated with 53 other features related to age, gender, ethnicity, language, ward, primary diagnosis, and delirium condition (see Additional files 1, 2 section for details). Particularly, sertraline prescriptions were positively correlated with VaD (correlation coefficient: 0.435), number of patients with depression indicated in admitting information (0.4535). Additionally, sertraline prescriptions were also positively correlated with the number of prescriptions of diazepam during admission. This pattern of drug prescription was also found during hospital stay. However, at the time of discharge, in terms of drug prescriptions, sertraline prescription was positively correlated with the number of diazepam prescriptions (0.4387), and mirtazapine prescriptions at the time of admission. After that, we investigated citalopram prescriptions; they were found to be positively correlated with the number of VaD (0.4107), bipolar patients (0.4028) at the time of admission. Additionally, they were positively related to valproate (0.4337) and diazepam (0.4168) prescriptions. However, during stay, citalopram prescriptions were related to VaD patients (0.4607) and venlafaxine (0.4388, at admission) and diazepam prescriptions (0.4778, 0.4198, at admission, and during stay respectively). This trend of prescription was similar at hospital discharge.

### Correlation between donepezil, memantine, sertraline, citalopram, risperidone and AD

We next investigated dementia drugs, particularly the number of donepezil prescriptions at the time of admission (Fig. 2, feature Ft184) and we observed that it was strongly correlated with 53 other features. Most of these features were similar to those for sertraline prescription, with some exceptions. For example, donepezil prescription number was not correlated with patient age (e.g. age group 66–80) nor the number of patients who spent the longest period with the surgery team. Further, donepezil prescription was weakly correlated with the number of VaD patients (0.0179) and other dementia subtypes except for AD (0.6643). Donepezil prescription was not highly correlated with co-prescription of antidepressants (e.g. citalopram 0.1469, 0.1750, 0.1874; sertraline 0.1571, 0.0948, 0.1086 at admission, during hospital stay and at discharge respectively). The number of donepezil prescription was more strongly correlated with AD patients during hospitalisation (0.6746) (Fig. 2, feature Ft68) and with the number of mirtazapine prescriptions at the time of hospital admission and during hospital stay (Fig. 2, feature Ft164 and 216, with value 0.4310 and 0.4248 respectively). Upon hospital discharge (Fig. 2, feature Ft286), we observed that donepezil prescription was correlated with 50 other features which were largely the same as those during hospital admission and stay (see Additional files 1, 2 section for details). Finally, we showed that memantine prescription numbers during admission, stay and discharge were (Ft 186, Ft238, and Ft288) positively correlated to number of AD patients (0.5782, 0.6049, and 0.6235). Additionally, its prescriptions during hospital stay and at discharge (Ft238, Ft288) were positively correlated with risperidone prescription number during hospital stay (Ft 198) (0.4857, 0.4069 respectively).

In summary, sertraline and citalopram prescriptions were positively correlated with VaD patients. Compared to citalopram, sertraline prescriptions were highly correlated with depressed patients whereas citalopram prescriptions were more correlated with bipolar patients only at the time of admission. Additionally, both these drugs were highly correlated with diazepam prescriptions except when citalopram was prescribed during discharge. In terms of dementia drugs, donepezil was highly correlated with mirtazapine prescriptions during hospital stay and discharge, whereas memantine prescriptions were more correlated with risperidone prescriptions during hospital stay.

Although the results based on correlation coefficients were substantial, they were limited to pairwise relationships. Hence, we next investigated simultaneous relationships among several of these features.

### Relationships among AD with prescribed dementia, antidepressant, and antipsychotic drugs

Building on our above correlation analysis of dementia subtypes with other features, we selected from the first 289 features which were related to the patient’s age, ethnicity, longest stay in the hospital, and medications at the time of admission, during hospital and discharge (see Fig. 2 caption for details). Then, we used multiple (linear) regression (Aiken et al. [34]) on the standardised dataset to investigate the relationships among antidepressant, antipsychotic, antianxiety, anticonvulsant, dementia drugs, depression and dementia subtypes (VaD, AD, FtD, and Parkinson's disease) (Methods), particularly on the prescribed drugs in each of these dementia subtypes (Additional file 4).

We first considered the number of donepezil prescriptions during hospital admission, stay and at discharge, and we found it was linked to the number of AD patients (R^2^: 0.4413, p-value: 1.4398E−07, R^2^: 0.4551, p-value: 7.7876E−08, and R^2^: 0.4018, p-value: 7.7583E−07 respectively) (for details, see Additional file 6: Tables S1). This was an expected result, validating the approach. During hospital stay, these prescriptions were significantly linked to mirtazapine prescriptions (R^2^: 0.9237, p-value: 1.0575E−25) (Additional file 6: Tables S2).

Similar to donepezil, the number of memantine prescriptions was (albeit weaker) positively dependent on AD patient number during hospital admission, stay and upon discharge (R^2^: 0.3344, p-value: 1.0933E−05, R^2^: 0.3659, p-value: 3.2715E−06, and R^2^: 0.3887, p-value: 1.3213E−06 respectively) (Additional file 6: Table S3) but showed no association with the number of donepezil prescriptions. Again, this was expected as they were not prescribed together. However, in the case of hospitalisation, the number of memantine prescriptions was very positively associated with risperidone prescriptions during stay and memantine prescriptions during admission (R^2^: 0.9469, p-value: 1.1136E−30) (Additional file 6: Table S4). Interestingly, such a memantine-risperidone association was missing at the time of discharge (Additional file 6: Table S4).

### Relationships among dementia subtypes (AD, VaD, and FtD), depressed patients with prescribed dementia, antidepressant, antipsychotic, mood stabiliser and antianxiety drugs

In terms of antidepressants, we first considered the number of sertraline prescriptions at admission, during hospital stay, and upon discharge, and observed that they were related to the number of VaD and depressed patients (R^2^: 0.2844, *p* value: 3.8438E−04, R^2^: 0.291, *p* value: 3.0947E−04, and R^2^: 0.2971, *p* value: 2.5274E−04 respectively) (Fig. 3A). (Additional file 6: Table S5). Additionally, sertraline prescription was also associated with the number of VaD patients and prescription of the anxiolytic drug diazepam at admission, during hospital stay, and at discharge (R^2^: 0.2834, *p* value: 3.9686E−04, R^2^: 0.2972, *p* value: 2.5142E−04, and R^2^: 0.2913, *p* value: 3.0650E−04 respectively) (Fig. 3B). (Additional file 6: Table S6). Also, sertraline prescriptions were associated with depression and diazepam prescriptions during discharge (R^2^: 0.3067, *p* value: 1.8257E−04) (Additional file 6: Table S7). This was expected given the co-morbidity of depression and anxiety.

Compared to the number of prescriptions of sertraline, the number of citalopram prescriptions on admission are related to diazepam and valproate prescriptions (R^2^- 3167, *p* value- 1.2987E−04) (Additional file 6: Table S8) (Fig. 3C). We further noticed that citalopram prescriptions during hospital stay were strongly associated with diazepam and citalopram prescriptions (R^2^- 0.9378, *p* value- 4.5418E−29) (Additional file 6: Table S9), but such trend was missing upon discharge. Hence, it was unclear whether the co-morbidity between depression and anxiety had been reduced upon discharge.

We next investigated the antidepressant Venlafaxine. We first observed that on admission, during hospital stay and upon discharge, venlafaxine is associated with the number of VaD and FtD patients but not AD patients (R^2^: 0.3184, *p* value: 1.2245E−04, R^2^: 0.3396, *p* value: 5.8312E−05, and R^2^: 0.3292, *p* value: 8.4114E−05 respectively) (Additional file 6: Table S10) (Fig. 3D). However, venlafaxine prescriptions during hospitalisation were strongly related to that during admission and citalopram prescriptions during hospital stay (r-squared- 0.9263, *p* value*p* value- 2.4024E−27) (Additional file 6: Table S11). Thus, both antidepressants were used during hospital stay.

We next looked at another antidepressant, mirtazapine. We noticed that on admission, during hospital stay and on discharge, the number of prescriptions of mirtazapine was associated with FtD patients (R^2^: 0.2299, *p* value: 4.2607E−04, R^2^: 0.2449, *p* value: 2.5921E−04, and R^2^: 0.2493, *p* value: 2.2299E−04 respectively) (Additional file 6: Table S12).

We then investigated the effects of the antiepileptic/anticonvulsant drug Lamotrigine. During hospital admission lamotrigine prescriptions was related to FtD patients, and number of valproate (mood stabiliser), and dosulepin (antidepressant) prescriptions (R^2^- 0.4684, *p* value- 1.8808E−06) (Additional file 6: Table S13). During hospitalisation, lamotrigine prescription was linked to FtD patients, but also to lamotrigine prescriptions on admission (R^2^- 0.5568, *p* value- 4.9519E−09) (Additional file 6: Table S14). On discharge, lamotrigine prescription relates to its hospitalisation prescriptions (R^2^- 0.7964, *p* value- 3.3073E−18) (Additional file 6: Table S15). Thus, lamotrigine had been prescribed on FtD patients throughout the hospitalisation journey.

Finally, we investigated the atypical antipsychotic, particularly quetiapine. Unlike the antidepressant mirtazapine, the number of quetiapine prescriptions during admission was associated with AD and not FtD patients (R^2^- 0.162, *p* value- 3.7537E−03) (Additional file 6: Table S16). During hospitalisation, quetiapine was only linked to its prescriptions on admission (R^2^- 0.8476, *p* value- 3.0343E−21) (Additional file 6: Table S17). However, on discharge, quetiapine was associated with the number of AD patients and previous quetiapine prescriptions (during admission and hospitalisations) (R^2^- 0.95, *p* value- 6.4821E−30) (Additional file 6: Table S18). This indicated the prescription of quetiapine prescriptions was associated with AD patients only during the hospital admission and during the discharge.

**Fig. 4:**
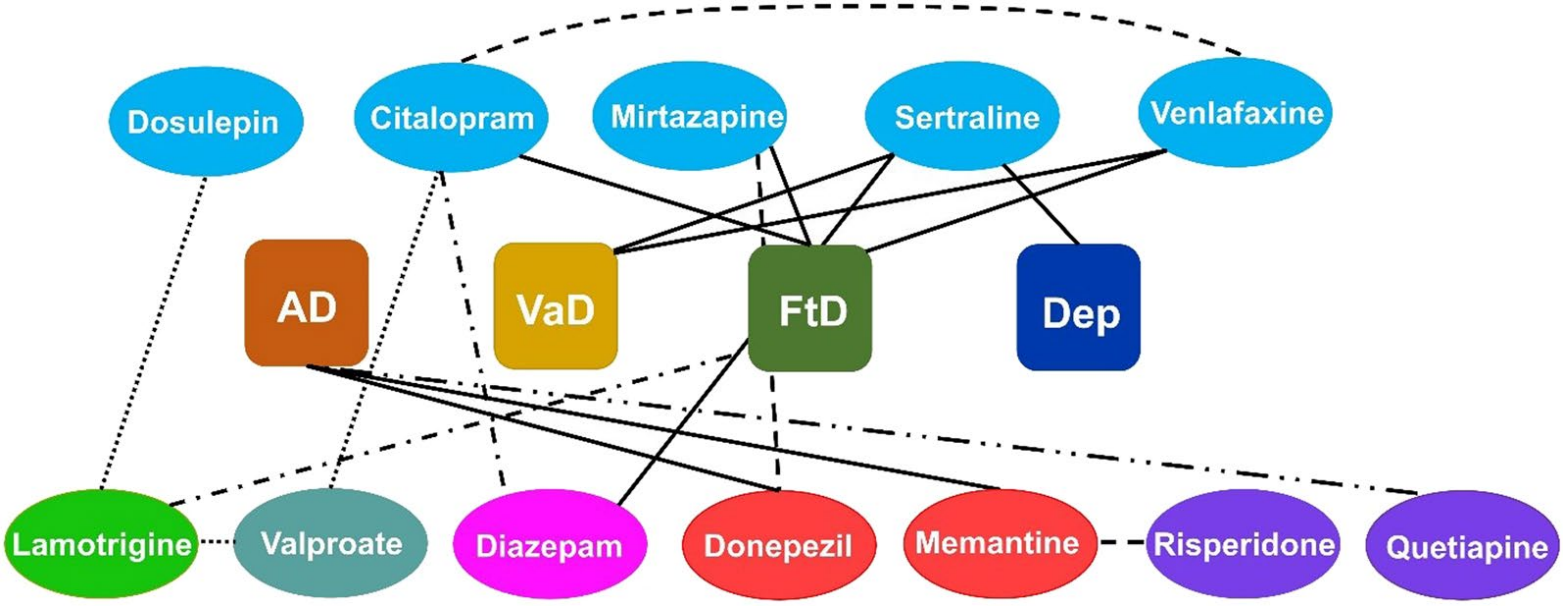
Association of drugs with Alzheimer’s disease (AD), vascular dementia (VaD), and depression (Dep). Six classes of drugs: antidepressants (blue), antipsychotic (violet), antianxiety (magenta), mood stabiliser (bluish grey), antiepileptic/ anticonvulsant (bright green) and dementia (red). Solid line indicates association during admission, stay and discharge, round dotted line denotes association during admission only, dashed line denotes association during hospital stay only, dash dotted line represents association between admission and stay, and dash double dotted line indicates association of number of prescriptions during admission and discharge. Note: Association among prescriptions of specific drugs during discharge with stay and admission, and association between drug prescription during hospital stay with admission are not shown in this figure, for details see (Additional files, Table S1–S18)

In summary, we had investigated the associations among antidepressant, mood stabilising, antipsychotic, antianxiety and dementia drugs, depression, and dementia subtypes, and how these were dependent on hospitalisation stage. In particular, we observed that the number of prescriptions of specific antidepressants (e.g. sertraline) was associated with the number of VaD patients, patients with depression and diazepam prescriptions. Likewise, the number of prescriptions for other antidepressant, citalopram was associated with diazepam prescriptions and venlafaxine was associated with VaD patients. In contrast, the antidepressant mirtazapine, was associated with other dementia subtypes (e.g. FtD patients) as well number of donepezil prescriptions**.** Further, some of these drugs showed multiple associations. For example, the number of lamotrigine (anticonvulsant) prescriptions was associated with FtD patients, valproate and dosulepin prescriptions. Additionally, the dementia drug memantine demonstrated multiple association with atypical antipsychotic drug risperidone and previous memantine prescriptions. A summary of the main results is illustrated in Fig. 4. These results are useful.

## Discussion

Dementia patients often live with BPSD and many comorbidities. Conventionally, antipsychotic drugs are used to treat BPSD related symptoms [38]. On the one hand, these drugs provide symptomatic relief to patients. On the other hand, they are often accompanied by undesirable side effects. Thus, many clinicians prefer to use antidepressants and mood stabilisers as a substitute for the treatment of BPSD [39]. Additionally, acetylcholinesterase inhibitors (donepezil) and memantine have been shown to positively influence the management of BPSD [40–42].

Multi-drug regimens are also a common strategy used in dementia patients to manage BPSD and treat co-morbid conditions [43–47]. Generally, a combination of drugs, widely known as polypharmacy, are prescribed to provide symptomatic relief, but a cocktail of these drugs often possesses a risk of adverse side-effects that may arise due to complex pharmacokinetics/pharmacodynamics and drug-drug interactions [48, 49]. The combination of drugs is prescribed after careful clinical assessment of medical history, longitudinal changes in behavioural and psychological symptoms, along with pathological imaging results [50, 51]. Therefore, prescription of drugs depends upon multiple factors. However, there is lack of a holistic understanding regarding prescription behaviours in actual clinical practice, especially in relation to patients’ hospital admission, during hospital in-patient stays, and upon discharge from hospital.

In this work, we aimed to identify multi-dimensional patterns in the prescription practice for antidepressant, antipsychotic, mood stabilising, anticonvulsant, and dementia treatment drugs by analysing the NAD data for hospitals in England and Wales. We were specifically interested in understanding the association among two or more data features involving drugs used in the treatment of dementia and BPSD including antidepressants, antipsychotics and anxiolytics and how these were associated with different subtypes of dementia and depression on hospital admission, during in-patient hospital stays and upon discharge. First, we applied correlation analysis to elucidate pairwise relationships between any two features. We then selected the features which are highly correlated with dementia subtypes (AD, VaD, and FtD). After that, we identified the drugs which are highly correlated with the above dementia subtypes, and then identified the features that are highly correlated with those drugs. We then manually selected (289) relevant features from the dataset. Finally, we conducted multiple linear regression analysis on the selected standardised features, and examined the relationships among polypharmacy, dementia subtypes, and depression.

Our analytical results on the NAD data identified several associations among drug prescriptions, admission, stay and discharge from hospitals, dementia subtypes and depression. For example, we identified a positive association between the number of sertraline prescriptions (during admission, stay and discharge) with the number of VaD patients and patients with depression. In practice, sertraline is a commonly prescribed drug for the treatment of depression in AD, although there are mixed results in the literature. For example, one study suggests that sertraline prescription can increase the likelihood of adverse effects in AD patients having depressive symptoms [52]. We also observed that the number of prescriptions of sertraline did not show any association with the number of prescriptions of the dementia treatment drug, donepezil. However, a study by Kumar and colleague suggests their co-administration to be safe in elderly patients [53]. In addition, on hospital admission, during hospital stay and on discharge, sertraline prescriptions were associated with the number of VaD patients and diazepam prescriptions. This is in contrast with earlier studies [54, 55] that suggest that sertraline administration can decrease the clearance of diazepam in plasma [54], suggesting that when the two drugs are prescribed together, the efficacy of diazepam can decrease. Nevertheless, in the absence of patient level detail, it is difficult to conclude their co-prescription is common, and more confirmatory studies are needed.

We also found that the number of citalopram prescriptions during admission was positively associated with diazepam and valproate prescriptions. Diazepam use is common in patients having BPSD symptoms [56], but usage of diazepam together with citalopram can increase the risk of side effects and may contribute to fatal poisoning [57]. In comparison, valproate can be effective in treating aggression and behavioural activation related symptoms [58], and its combination with citalopram has proven to be effective, especially in depressed patients with dysphoric mood [58]

Additionally, during hospitalisation, we observed that the prescription of antidepressant venlafaxine was highly associated with citalopram prescriptions. Both these drugs are primarily used to reduce anxiety in dementia patients [59]. In depressed patients, their efficacies are the same when there is insufficient response to other antidepressants [60]. However, for a group of depressed patients, venlafaxine is found to be therapeutically more effective than other SSRIs, as it enhances baseline brain levels of both serotonin and norepinephrine [60]. Further, co-prescription of both these drugs can result in adverse side effects (e.g. dizziness or agitation) and increase healthcare prescription cost [61]. However, it is difficult to conclude, at the coarse-grained hospital level as in this study, whether the same patients were co-prescribed citalopram and venlafaxine. Future work will analyse at patient-level detail.

In terms of atypical antipsychotics, we found that the number of prescriptions of quetiapine was associated with the number of AD patients during admission. Another commonly prescribed atypical antipsychotic drug, risperidone, is also suggested to be an effective medication for BPSD [62]. However, due to its adverse effects including on the cerebrovascular system, its usage in elderly patients is still debatable in clinical practice in many countries [62]. Generally, low dosage of quetiapine is as equally effective as risperidone and is tolerated well by elderly patients with BPSD [63].

Finally, we demonstrated that the number of prescriptions of donepezil was positively associated with the number of AD patients and mirtazapine prescriptions. However, donepezil prescriptions did not show any association with the number of memantine prescriptions which is not consistent with other works that suggest that co-administration of memantine considerably improves the mental health in moderate AD patients who are already on donepezil [64, 65]. This discrepancy in prescriptive behaviour and empirical research points towards the need for further empirical evidence and also the analysis of more granular data (e.g. at the patient level). These results could be useful in understanding clinicians’ prescribing behaviour, for instance, in understanding whether specific drug prescribed during admission is discontinued during hospitalisation or discharge.

## Conclusion

Our results demonstrate the complex, multi-dimensional relationship between polypharmacy, dementia subtypes and neuropsychiatric disorders. We found that the prescription of drugs is influenced by dementia subtype, the presence of depression, the prescription of other (antidepressant, antipsychotic, mood stabilising, anticonvulsant, and dementia) drugs on admission to hospital, prescription during in-patient hospital stays or on discharge. Most importantly, our work captures the relationship among commonly prescribed drugs, and could be useful in unfolding drug co-prescription patterns. We believe such approaches will assist in decision making and allow clinicians and healthcare planners to better evaluate the costs and benefits of polypharmacy.

## Supplementary Information

**Additional file 1.** Correlation among the features.

**Additional file 2.** Correlation values exceed the Pearson pairwise correlation coefficient threshold of 0.4.

**Additional file 3.** NAD dataset.

**Additional file 4.** Features used for association study.

**Additional file 5.** Standardised features.

**Additional file 6.** Dataset details and supplementary results that explore the relationships among dementia, depression, and prescribed drugs.

## Data Availability

This study did not require ethical approval as analysis was performed on the open National Audit of Dementia (NAD) available at [https://www.rcpsych.ac.uk/improving-care/ccqi/national-clinical-audits/national-audit-of-dementia/nad-reports-and-resources/data-tables#faq-accoridon-collapse2563078b-adf0-4aa7-83ae-b771666af179]. All data generated or analysed during this study are included in this published article [and its supplementary information files.
