# Supplementary material for: Multi-dimensional relationships among dementia, depression and prescribed drugs in England and Wales hospitals
Source: BMC Med Inform Decis Mak. 2022 Oct 7;22:262. doi: 10.1186/s12911-022-01892-9 (PMC9547465; doi:10.1186/s12911-022-01892-9)
Supplement: Supplementary file 4 — Additional file 4. Features used for association study. [file 12911_2022_1892_MOESM4_ESM.docx]

**Dementia subtypes:** correlations with other features, violet: drug related features considered in this analysis.

**Dementia AD**

'N_BREAK' 'Q1_Age_Recoded_D' 'Q1_Age_Recoded_num_66to80' 'Q1_Age_Recoded_num_81to100' 'Q2_Gender_D' 'Q2_Gender_num_female' 'Q2_Gender_num_male' 'Q3_Ethnicity_Recoded_D' 'Q4_Language_Recoded_D' 'Q4_Language_Recoded_num_English' 'Q5_Ward_Recoded_D' 'Q5_Ward_Recoded_num_Surgical' 'Q6_Prim_diagnosis_D' 'Q6_Prim_diagnosis_num_respiratory' 'Q7_Del_on_adm_D' 'Q7_no_Del_on_adm' 'Q8_Del_dur_adm_D' 'Q8_no_Del_dur_adm' 'Q9_dem_subtype_D' 'Q9b_Mixed_atypical_D' 'Q9b_Mixed_atypical_no_num' 'Q10_ICD_10' 'Q10_10_none_num' 'Q11_died_D' 'Q11_died_no_num' 'Q12_self_disc_D' 'Q12_self_disc_no_num' 'Q13_fast_track_D' 'Q13_fast_track_no_num' 'Q14_EOL_D' 'Q14_EOL_no_num' 'LOS_Range_D' 'LOS_Range_num_1to10' 'LOS_Range_num_11to20' 'Q17_res_before_D' 'Q17_res_before_num_Own' 'Q17_res_before_num_Residential' 'Q18_res_after_D' 'Q18_res_after_num_Own' 'Q18_res_after_num_Residential' 'S2A_Quetiapine_num' 'Q25_1_Donepezil_num' 'Q25_3_Memantine_num' 'Q28_22_Quetiapine_num' 'Q32_1_Donepezil_num' 'Q32_3_Memantine_num' 'Q35_22_Quetiapine_num' 'Q39_1_Donepezil_num' 'Q39_3_Memantine_num' 'drugsequence_A_5_8_presc_num' 'drugsequences5_8_3_10_6_11_7_9_presc_num' 'drugsequenceALLtotalexclpromethlith_presc_D' 'drugsequence_A_5_8_hypnotics_presc_num' 'drugsequence_A_5_8_antidep_presc_num' 'drugsequence_A_5_8_dem_drugs_presc_num' 'drugsequences5_8_3_10_6_11_7_9_antidep_presc_num' 'drugsequences5_8_3_10_6_11_7_9_dem_drugs_presc_num' 'drugsequences5_8_3_10_7_9_presc_num' 'drugsequences_demdrugs_5_8_3_10_7_9_presc_D' 'Antidepressants_reg_pres_presc_num' 'Dementia_med_reg_pres_presc_num' 'All_classes_2A_prescription_countpresc_den' 'All_classes_2B_prescription_countpresc_den' 'All_classes_2C_prescription_countpresc_den' 'Antidepressants2A_Count_presc_num' 'DrugsDem2A_Count_presc_num' 'Antidepressants2B_Count_presc_num' 'DrugsDem2B_Count_presc_num' 'DrugsDem2C_Count_presc_num' 'Dementia_med_review_presc_num' 'Dementia_med_review_rec_presc_num'

**Parkinsons**

'Q1_Age_min' 'Q7_Del_on_adm_num' 'Q8_Del_dur_adm_num' 'Q23_9_Escitalopram_num'

**Frontotemporal**

'Q6_Prim_diagnosis_num_Fall' 'LOS_Range_num_31to40' 'Q18_res_after_num_transfer' 'Q22_2_Lamotrigine_num' 'Q23_16_Mirtazapine_num' 'Q23_30_Venlafaxine_num' 'Q29_2_Lamotrigine_num' 'Q30_16_Mirtazapine_num' 'Q30_30_Venlafaxine_num' 'Q36_2_Lamotrigine_num' 'Q37_16_Mirtazapine_num' 'Q37_27_Trazodone_num' 'Q37_30_Venlafaxine_num' 'drugsequence_A_5_8_antidep_presc_num' 'drugsequences5_8_3_10_6_11_7_9_antidep_presc_num' 'drugsequences_antidep_5_8_3_10_7_9_presc_D' 'Antidepressants_reg_pres_presc_num' 'Antidepressants2A_Count_presc_num' 'Antidepressants2B_Count_presc_num' 'Antidepressants2C_Count_presc_num' 'Antidep_new_pres_symptoms_rec_sleep_disturbance_presc_num' 'Antidepressant_review_rec_presc_num' 'Antidep_new_prescriptions_recommended_for_review_presc_num'

**Vascular dementia**

'N_BREAK' 'Q1_Age_Recoded_D' 'Q1_Age_Recoded_num_66to80' 'Q1_Age_Recoded_num_81to100' 'Q2_Gender_D' 'Q2_Gender_num_female' 'Q2_Gender_num_male' 'Q3_Ethnicity_Recoded_D' 'Q3_Ethnicity_Recoded_num_white' 'Q4_Language_Recoded_D' 'Q5_Ward_Recoded_D' 'Q6_Prim_diagnosis_D' 'Q7_Del_on_adm_D' 'Q7_no_Del_on_adm' 'Q8_Del_dur_adm_D' 'Q8_no_Del_dur_adm' 'Q9_dem_subtype_D' 'Q10_ICD_10' 'Q10_7_yes_schizophrenia_num' 'Q11_died_D' 'Q11_died_no_num' 'Q12_self_disc_D' 'Q12_self_disc_no_num' 'Q13_fast_track_D' 'Q13_fast_track_no_num' 'Q14_EOL_D' 'Q14_EOL_no_num' 'LOS_Range_D' 'LOS_Range_num_1to10' 'Q17_res_before_D' 'Q17_res_before_num_Nursing' 'Q17_res_before_num_Own' 'Q18_res_after_D' 'Q23_4_Citalopram_num' 'Q23_25_Sertraline_num' 'Q23_30_Venlafaxine_num' 'Q30_2_Amitriptyline_num' 'Q30_4_Citalopram_num' 'Q30_25_Sertraline_num' 'Q30_30_Venlafaxine_num' 'Q37_4_Citalopram_num' 'Q37_25_Sertraline_num' 'Q37_30_Venlafaxine_num' 'drugsequence_A_5_8_presc_num' 'drugsequences5_8_3_10_6_11_7_9_presc_num' 'drugsequenceALLtotalexclpromethlith_presc_D' 'drugsequence_A_5_8_antipsychotics_presc_num' 'drugsequence_A_5_8_antidep_presc_num' 'drugsequences5_8_3_10_6_11_7_9_antipsychotics_presc_num' 'drugsequences5_8_3_10_6_11_7_9_antidep_presc_num' 'drugsequences5_8_3_10_7_9_presc_num' 'drugsequences_antidep_5_8_3_10_7_9_presc_D' 'drugsequences_hypnotics_5_8_3_10_7_9_presc_D' 'Antidepressants_reg_pres_presc_num' 'All_classes_2A_prescription_countpresc_den' 'All_classes_2B_prescription_countpresc_den' 'All_classes_2C_prescription_countpresc_den' 'Antipsychotics2A_Count_presc_num' 'Antidepressants2A_Count_presc_num' 'Antidepressants2B_Count_presc_num' 'Hypnotics2B_Count_presc_num' 'Antidepressants2C_Count_presc_num' 'Antidepressant_review_rec_presc_num'

**Drugs features:** extracted and further explored for correlation from above dementia subtypes, violet: features selected for association analysis, black: features ignored.

**'S2A_Quetiapine_num'**

'Q6_Prim_diagnosis_num_Endocrine' 'Q9_dem_subtype_num_Alzheimers' 'Q9b_Mixed_atypical_D' 'Q9b_Mixed_atypical_no_num' 'Q28_22_Quetiapine_num' 'Q35_22_Quetiapine_num' 'drugsequence_A_5_8_antipsychotics_presc_num' 'drugsequences5_8_3_10_6_11_7_9_antipsychotics_presc_num' 'drugsequences_antipsych_5_8_3_10_7_9_presc_D' 'Antipsychotics_reg_pres_presc_num' 'Antipsychotics2A_Count_presc_num' 'Antipsychotics2B_Count_presc_num' 'Antipsychotics2C_Count_presc_num' 'Antipsychotics_review_presc_num' 'Antipsychotics_review_rec_presc_num'

**'Q25_1_Donepezil_num'**

'N_BREAK' 'Q1_Age_Recoded_D' 'Q1_Age_Recoded_num_81to100' 'Q2_Gender_D' 'Q2_Gender_num_male' 'Q3_Ethnicity_Recoded_D' 'Q4_Language_Recoded_D' 'Q4_Language_Recoded_num_English' 'Q5_Ward_Recoded_D' 'Q6_Prim_diagnosis_D' 'Q6_Prim_diagnosis_num_respiratory' 'Q7_Del_on_adm_D' 'Q7_no_Del_on_adm' 'Q8_Del_dur_adm_D' 'Q8_no_Del_dur_adm' 'Q9_dem_subtype_D' 'Q9_dem_subtype_num_Alzheimers' 'Q9b_Mixed_atypical_D' 'Q9b_Mixed_atypical_yes_num' 'Q9b_Mixed_atypical_no_num' 'Q10_ICD_10' 'Q11_died_D' 'Q11_died_no_num' 'Q12_self_disc_D' 'Q12_self_disc_no_num' 'Q13_fast_track_D' 'Q13_fast_track_no_num' 'Q14_EOL_D' 'Q14_EOL_no_num' 'LOS_Range_D' 'Q17_res_before_D' 'Q17_res_before_num_Own' 'Q18_res_after_D' 'Q18_res_after_num_Own' 'Q32_1_Donepezil_num' 'Q39_1_Donepezil_num' 'drugsequence_A_5_8_presc_num' 'drugsequences5_8_3_10_6_11_7_9_presc_num' 'drugsequenceALLtotalexclpromethlith_presc_D' 'drugsequence_A_5_8_dem_drugs_presc_num' 'drugsequences5_8_3_10_6_11_7_9_dem_drugs_presc_num' 'drugsequences5_8_3_10_7_9_presc_num' 'drugsequences_demdrugs_5_8_3_10_7_9_presc_D' 'Dementia_med_reg_pres_presc_num' 'All_classes_2A_prescription_countpresc_den' 'All_classes_2B_prescription_countpresc_den' 'All_classes_2C_prescription_countpresc_den' 'DrugsDem2A_Count_presc_num' 'DrugsDem2B_Count_presc_num' 'DrugsDem2C_Count_presc_num' 'Demdrug_new_prescriptions_recby_ward_presc_num' 'Dementia_med_review_presc_num' 'Dementia_med_review_rec_presc_num'

**23_25 Sertraline**

'N_BREAK' 'Q1_Age_Recoded_D' 'Q1_Age_Recoded_num_66to80' 'Q2_Gender_D' 'Q2_Gender_num_female' 'Q3_Ethnicity_Recoded_D' 'Q4_Language_Recoded_D' 'Q5_Ward_Recoded_D' 'Q5_Ward_Recoded_num_Surgical' 'Q6_Prim_diagnosis_D' 'Q7_Del_on_adm_D' 'Q8_Del_dur_adm_D' 'Q8_no_Del_dur_adm' 'Q9_dem_subtype_D' 'Q9_dem_subtype_num_Vascular' 'Q10_ICD_10' 'Q10_2_yes_depress_num' 'Q10_10_none_num' 'Q11_died_D' 'Q11_died_no_num' 'Q12_self_disc_D' 'Q12_self_disc_no_num' 'Q13_fast_track_D' 'Q13_fast_track_no_num' 'Q14_EOL_D' 'Q14_EOL_no_num' 'LOS_Range_D' 'LOS_Range_num_1to10' 'Q17_res_before_D' 'Q17_res_before_num_Nursing' 'Q18_res_after_D' 'Q24_9_Diazepam_num' 'Q30_25_Sertraline_num' 'Q31_9_Diazepam_num' 'Q37_25_Sertraline_num' 'Q38_9_Diazepam_num'

**'Q25_3_Memantine_num'**

'N_BREAK' 'Q1_Age_Recoded_D' 'Q1_Age_Recoded_num_66to80' 'Q1_Age_Recoded_num_81to100' 'Q2_Gender_D' 'Q2_Gender_num_female' 'Q2_Gender_num_male' 'Q3_Ethnicity_Recoded_D' 'Q3_Ethnicity_Recoded_num_white' 'Q4_Language_Recoded_D' 'Q5_Ward_Recoded_D' 'Q5_Ward_Recoded_num_Other' 'Q6_Prim_diagnosis_D' 'Q6_Prim_diagnosis_num_Cardiac' 'Q6_Prim_diagnosis_num_Dementia' 'Q6_Prim_diagnosis_num_respiratory' 'Q7_Del_on_adm_D' 'Q7_no_Del_on_adm' 'Q8_Del_dur_adm_D' 'Q8_no_Del_dur_adm' 'Q9_dem_subtype_D' 'Q9_dem_subtype_num_Alzheimers' 'Q9b_Mixed_atypical_D' 'Q9b_Mixed_atypical_no_num' 'Q10_ICD_10' 'Q10_10_none_num' 'Q11_died_D' 'Q11_died_no_num' 'Q12_self_disc_D' 'Q12_self_disc_no_num' 'Q13_fast_track_D' 'Q13_fast_track_no_num' 'Q14_EOL_D' 'Q14_EOL_no_num' 'LOS_Range_D' 'LOS_Range_num_1to10' 'LOS_Range_num_11to20' 'Q17_res_before_D' 'Q17_res_before_num_Own' 'Q18_res_after_D' 'Q18_res_after_num_Own' 'Q18_res_after_num_Residential' 'Q32_3_Memantine_num' 'Q39_3_Memantine_num' 'drugsequence_A_5_8_presc_num' 'drugsequences5_8_3_10_6_11_7_9_presc_num' 'drugsequenceALLtotalexclpromethlith_presc_D' 'drugsequence_A_5_8_antipsychotics_presc_num' 'drugsequence_A_5_8_dem_drugs_presc_num' 'drugsequences5_8_3_10_6_11_7_9_dem_drugs_presc_num' 'drugsequences5_8_3_10_7_9_presc_num' 'drugsequences_demdrugs_5_8_3_10_7_9_presc_D' 'Dementia_med_reg_pres_presc_num' 'All_classes_2A_prescription_countpresc_den' 'All_classes_2B_prescription_countpresc_den' 'All_classes_2C_prescription_countpresc_den' 'DrugsDem2A_Count_presc_num' 'Antipsychotics2B_Count_presc_num' 'DrugsDem2B_Count_presc_num' 'DrugsDem2C_Count_presc_num' 'Dementia_med_review_presc_num' 'Dementia_med_review_rec_presc_num'

**'Q28_22_Quetiapine_num'**

'Q9_dem_subtype_num_Alzheimers' 'Q9b_Mixed_atypical_D' 'Q9b_Mixed_atypical_no_num' 'Q10_1_yes_bipolar_num' 'Q17_res_before_num_Residential' 'Q18_res_after_num_Residential' 'S2A_Quetiapine_num' 'Q35_22_Quetiapine_num' 'drugsequence_A_5_8_antipsychotics_presc_num' 'drugsequences5_8_3_10_6_11_7_9_antipsychotics_presc_num' 'drugsequences_antipsych_5_8_3_10_7_9_presc_D' 'Antipsychotics_reg_pres_presc_num' 'Antipsychotics2A_Count_presc_num' 'Antipsychotics2B_Count_presc_num' 'Antipsychotics2C_Count_presc_num' 'Antipsychotics_review_presc_num' 'Antipsychotics_review_rec_presc_num'

**'Q32_1_Donepezil_num'**

'N_BREAK' 'Q1_Age_Recoded_D' 'Q1_Age_Recoded_num_81to100' 'Q2_Gender_D' 'Q2_Gender_num_male' 'Q3_Ethnicity_Recoded_D' 'Q4_Language_Recoded_D' 'Q5_Ward_Recoded_D' 'Q6_Prim_diagnosis_D' 'Q6_Prim_diagnosis_num_respiratory' 'Q7_Del_on_adm_D' 'Q7_no_Del_on_adm' 'Q8_Del_dur_adm_D' 'Q8_no_Del_dur_adm' 'Q9_dem_subtype_D' 'Q9_dem_subtype_num_Alzheimers' 'Q9b_Mixed_atypical_D' 'Q9b_Mixed_atypical_no_num' 'Q10_ICD_10' 'Q11_died_D' 'Q11_died_no_num' 'Q12_self_disc_D' 'Q12_self_disc_no_num' 'Q13_fast_track_D' 'Q13_fast_track_no_num' 'Q14_EOL_D' 'Q14_EOL_no_num' 'LOS_Range_D' 'Q17_res_before_D' 'Q17_res_before_num_Own' 'Q18_res_after_D' 'Q23_16_Mirtazapine_num' 'Q25_1_Donepezil_num' 'Q30_16_Mirtazapine_num' 'Q37_16_Mirtazapine_num' 'Q39_1_Donepezil_num' 'drugsequence_A_5_8_presc_num' 'drugsequences5_8_3_10_6_11_7_9_presc_num' 'drugsequenceALLtotalexclpromethlith_presc_D' 'drugsequence_A_5_8_antidep_presc_num' 'drugsequence_A_5_8_dem_drugs_presc_num' 'drugsequences5_8_3_10_6_11_7_9_dem_drugs_presc_num' 'drugsequences5_8_3_10_7_9_presc_num' 'drugsequences_demdrugs_5_8_3_10_7_9_presc_D' 'Dementia_med_reg_pres_presc_num' 'All_classes_2A_prescription_countpresc_den' 'All_classes_2B_prescription_countpresc_den' 'All_classes_2C_prescription_countpresc_den' 'DrugsDem2A_Count_presc_num' 'DrugsDem2B_Count_presc_num' 'DrugsDem2C_Count_presc_num' 'Dementia_med_review_presc_num' 'Dementia_med_review_rec_presc_num'

**'Q32_3_Memantine_num'**

'N_BREAK' 'Q1_Age_Recoded_D' 'Q1_Age_Recoded_num_66to80' 'Q1_Age_Recoded_num_81to100' 'Q2_Gender_D' 'Q2_Gender_num_female' 'Q2_Gender_num_male' 'Q3_Ethnicity_Recoded_D' 'Q3_Ethnicity_Recoded_num_white' 'Q4_Language_Recoded_D' 'Q5_Ward_Recoded_D' 'Q6_Prim_diagnosis_D' 'Q6_Prim_diagnosis_num_Cardiac' 'Q6_Prim_diagnosis_num_Dementia' 'Q6_Prim_diagnosis_num_respiratory' 'Q7_Del_on_adm_D' 'Q7_no_Del_on_adm' 'Q8_Del_dur_adm_D' 'Q8_no_Del_dur_adm' 'Q9_dem_subtype_D' 'Q9_dem_subtype_num_Alzheimers' 'Q9b_Mixed_atypical_D' 'Q9b_Mixed_atypical_no_num' 'Q10_ICD_10' 'Q10_10_none_num' 'Q11_died_D' 'Q11_died_no_num' 'Q12_self_disc_D' 'Q12_self_disc_no_num' 'Q13_fast_track_D' 'Q13_fast_track_no_num' 'Q14_EOL_D' 'Q14_EOL_no_num' 'LOS_Range_D' 'LOS_Range_num_1to10' 'LOS_Range_num_11to20' 'Q17_res_before_D' 'Q17_res_before_num_Own' 'Q18_res_after_D' 'Q18_res_after_num_Own' 'Q18_res_after_num_Residential' 'Q25_3_Memantine_num' 'Q28_23_Risperidone_num' 'Q39_3_Memantine_num' 'drugsequence_A_5_8_presc_num' 'drugsequences5_8_3_10_6_11_7_9_presc_num' 'drugsequenceALLtotalexclpromethlith_presc_D' 'drugsequence_A_5_8_antipsychotics_presc_num' 'drugsequence_A_5_8_antidep_presc_num' 'drugsequence_A_5_8_dem_drugs_presc_num' 'drugsequences5_8_3_10_6_11_7_9_antipsychotics_presc_num' 'drugsequences5_8_3_10_6_11_7_9_antidep_presc_num' 'drugsequences5_8_3_10_6_11_7_9_dem_drugs_presc_num' 'drugsequences5_8_3_10_7_9_presc_num' 'drugsequences_antidep_5_8_3_10_7_9_presc_D' 'drugsequences_demdrugs_5_8_3_10_7_9_presc_D' 'drugsequences_antipsych_5_8_3_10_7_9_presc_D' 'Antipsychotics_reg_pres_presc_num' 'Antidepressants_reg_pres_presc_num' 'Dementia_med_reg_pres_presc_num' 'All_classes_2A_prescription_countpresc_den' 'All_classes_2B_prescription_countpresc_den' 'All_classes_2C_prescription_countpresc_den' 'Antipsychotics2A_Count_presc_num' 'Antidepressants2A_Count_presc_num' 'DrugsDem2A_Count_presc_num' 'Antipsychotics2B_Count_presc_num' 'Antidepressants2B_Count_presc_num' 'DrugsDem2B_Count_presc_num' 'Antipsychotics2C_Count_presc_num' 'Antidepressants2C_Count_presc_num' 'DrugsDem2C_Count_presc_num' 'Dementia_med_review_presc_num' 'Antipsychotics_review_rec_presc_num' 'Dementia_med_review_rec_presc_num' 'Antidepressant_review_rec_presc_num'

**'Q35_22_Quetiapine_num'**

**'Q9_dem_subtype_num_Alzheimers' 'Q9b_Mixed_atypical_D' 'Q9b_Mixed_atypical_no_num' 'Q18_res_after_num_Residential' 'S2A_Quetiapine_num' 'Q28_22_Quetiapine_num'** 'drugsequence_A_5_8_antipsychotics_presc_num' 'drugsequences5_8_3_10_6_11_7_9_antipsychotics_presc_num' 'drugsequences_antipsych_5_8_3_10_7_9_presc_D' 'Antipsychotics_reg_pres_presc_num' 'Antipsychotics2A_Count_presc_num' 'Antipsychotics2B_Count_presc_num' 'Antipsychotics2C_Count_presc_num' 'Antidep_new_prescriptions_recby_liaison_presc_num' 'Antipsychotics_review_presc_num' 'Antipsychotics_review_rec_presc_num'

**'Q39_1_Donepezil_num'**

'N_BREAK' 'Q1_Age_Recoded_D' 'Q1_Age_Recoded_num_81to100' 'Q2_Gender_D' 'Q2_Gender_num_male' 'Q3_Ethnicity_Recoded_D' 'Q4_Language_Recoded_D' 'Q5_Ward_Recoded_D' 'Q6_Prim_diagnosis_D' 'Q6_Prim_diagnosis_num_respiratory' 'Q7_Del_on_adm_D' 'Q7_no_Del_on_adm' 'Q8_Del_dur_adm_D' 'Q8_no_Del_dur_adm' 'Q9_dem_subtype_D' 'Q9_dem_subtype_num_Alzheimers' 'Q9b_Mixed_atypical_D' 'Q9b_Mixed_atypical_no_num' 'Q10_ICD_10' 'Q11_died_D' 'Q11_died_no_num' 'Q12_self_disc_D' 'Q12_self_disc_no_num' 'Q13_fast_track_D' 'Q13_fast_track_no_num' 'Q14_EOL_D' 'Q14_EOL_no_num' 'LOS_Range_D' 'Q17_res_before_D' 'Q17_res_before_num_Own' 'Q18_res_after_D' 'Q18_res_after_num_Own' 'Q25_1_Donepezil_num' 'Q32_1_Donepezil_num' 'drugsequence_A_5_8_presc_num' 'drugsequences5_8_3_10_6_11_7_9_presc_num' 'drugsequenceALLtotalexclpromethlith_presc_D' 'drugsequence_A_5_8_dem_drugs_presc_num' 'drugsequences5_8_3_10_6_11_7_9_dem_drugs_presc_num' 'drugsequences5_8_3_10_7_9_presc_num' 'drugsequences_demdrugs_5_8_3_10_7_9_presc_D' 'Dementia_med_reg_pres_presc_num' 'All_classes_2A_prescription_countpresc_den' 'All_classes_2B_prescription_countpresc_den' 'All_classes_2C_prescription_countpresc_den' 'DrugsDem2A_Count_presc_num' 'DrugsDem2B_Count_presc_num' 'DrugsDem2C_Count_presc_num' 'Dementia_med_review_presc_num' 'Dementia_med_review_rec_presc_num'

**'Q39_3_Memantine_num'**

'N_BREAK' 'Q1_Age_Recoded_D' 'Q1_Age_Recoded_num_66to80' 'Q1_Age_Recoded_num_81to100' 'Q2_Gender_D' 'Q2_Gender_num_female' 'Q2_Gender_num_male' 'Q3_Ethnicity_Recoded_D' 'Q3_Ethnicity_Recoded_num_white' 'Q4_Language_Recoded_D' 'Q4_Language_Recoded_num_English' 'Q5_Ward_Recoded_D' 'Q6_Prim_diagnosis_D' 'Q6_Prim_diagnosis_num_Cardiac' 'Q6_Prim_diagnosis_num_Dementia' 'Q6_Prim_diagnosis_num_respiratory' 'Q7_Del_on_adm_D' 'Q7_no_Del_on_adm' 'Q8_Del_dur_adm_D' 'Q8_no_Del_dur_adm' 'Q9_dem_subtype_D' 'Q9_dem_subtype_num_Alzheimers' 'Q9b_Mixed_atypical_D' 'Q9b_Mixed_atypical_no_num' 'Q10_ICD_10' 'Q10_10_none_num' 'Q11_died_D' 'Q11_died_no_num' 'Q12_self_disc_D' 'Q12_self_disc_no_num' 'Q13_fast_track_D' 'Q13_fast_track_no_num' 'Q14_EOL_D' 'Q14_EOL_no_num' 'LOS_Range_D' 'LOS_Range_num_1to10' 'LOS_Range_num_11to20' 'Q17_res_before_D' 'Q17_res_before_num_Own' 'Q18_res_after_D' 'Q18_res_after_num_Own' 'Q18_res_after_num_Residential' 'Q25_3_Memantine_num' 'Q28_23_Risperidone_num' 'Q32_3_Memantine_num' 'drugsequence_A_5_8_presc_num' 'drugsequences5_8_3_10_6_11_7_9_presc_num' 'drugsequenceALLtotalexclpromethlith_presc_D' 'drugsequence_A_5_8_antipsychotics_presc_num' 'drugsequence_A_5_8_antidep_presc_num' 'drugsequence_A_5_8_dem_drugs_presc_num' 'drugsequences5_8_3_10_6_11_7_9_antidep_presc_num' 'drugsequences5_8_3_10_6_11_7_9_dem_drugs_presc_num' 'drugsequences5_8_3_10_7_9_presc_num' 'drugsequences_antidep_5_8_3_10_7_9_presc_D' 'drugsequences_demdrugs_5_8_3_10_7_9_presc_D' 'Antipsychotics_reg_pres_presc_num' 'Antidepressants_reg_pres_presc_num' 'Dementia_med_reg_pres_presc_num' 'All_classes_2A_prescription_countpresc_den' 'All_classes_2B_prescription_countpresc_den' 'All_classes_2C_prescription_countpresc_den' 'Antidepressants2A_Count_presc_num' 'DrugsDem2A_Count_presc_num' 'Antipsychotics2B_Count_presc_num' 'Antidepressants2B_Count_presc_num' 'DrugsDem2B_Count_presc_num' 'Antipsychotics2C_Count_presc_num' 'Antidepressants2C_Count_presc_num' 'DrugsDem2C_Count_presc_num' 'Dementia_med_review_presc_num' 'Antipsychotics_review_rec_presc_num' 'Dementia_med_review_rec_presc_num' 'Antidepressant_review_rec_presc_num'

**'Q23_9_Escitalopram_num'**

'Q9_dem_subtype_num_Parkinsons' 'Q30_9_Escitalopram_num' 'Q37_9_Escitalopram_num' 'Antipsych_new_presc_2_4_recom_notrev_presc_num' 'Combined_new_presc_2_4_recom_notrev_presc_num' 'Antidepressants_prn_pres_presc_num' 'Antipsych_symptoms_aggression_presc_num' 'Demdrug_symptoms_other_presc_num' 'Hypnotics_symptoms_depression_presc_num' 'Demdrug_new_pres_symptoms_rec_other_symtpom_presc_num' 'Hypnotics_new_pres_symptoms_rec_depression_presc_num' 'Demdrug_new_prescriptions_recby_ward_presc_num'

'Q22_2_Lamotrigine_num'

'Q6_Prim_diagnosis_num_pain' 'Q9_dem_subtype_num_Frontotemporal' 'Q22_4_Valproate_num' 'Q23_6_Dosulepin_num' 'Q24_17_Nitrazepam_num' 'Q29_2_Lamotrigine_num' 'Q30_6_Dosulepin_num' 'Q31_17_Nitrazepam_num' 'Q36_2_Lamotrigine_num' 'Q38_17_Nitrazepam_num' 'drugsequence_A_5_8_anticonvulsants_presc_num' 'drugsequence_B_3_10_anticonvulsants_presc_num' 'drugsequence_C_6_11_anticonvulsants_presc_num' 'drugsequences5_8_3_10_6_11_7_9_anticonvulsants_presc_num' 'drugsequences_anticon_5_8_3_10_7_9_presc_D' 'Anticonvulsants_reg_pres_presc_num' 'Anticon2A_Count_presc_num' 'Anticon2C_Count_presc_num' 'Anticonvulsant_review_rec_presc_num'

**'Q23_16_Mirtazapine_num'**

'N_BREAK' 'Q1_Age_Recoded_D' 'Q1_Age_Recoded_num_66to80' 'Q1_Age_Recoded_num_81to100' 'Q1_Age_min' 'Q2_Gender_D' 'Q2_Gender_num_female' 'Q2_Gender_num_male' 'Q3_Ethnicity_Recoded_D' 'Q3_Ethnicity_Recoded_num_white' 'Q4_Language_Recoded_D' 'Q5_Ward_Recoded_D' 'Q6_Prim_diagnosis_D' 'Q6_Prim_diagnosis_num_respiratory' 'Q7_Del_on_adm_D' 'Q7_no_Del_on_adm' 'Q8_Del_dur_adm_D' 'Q8_no_Del_dur_adm' 'Q9_dem_subtype_D' 'Q9_dem_subtype_num_Frontotemporal' 'Q10_ICD_10' 'Q10_10_none_num' 'Q11_died_D' 'Q11_died_no_num' 'Q12_self_disc_D' 'Q12_self_disc_no_num' 'Q13_fast_track_D' 'Q13_fast_track_no_num' 'Q14_EOL_D' 'Q14_EOL_no_num' 'LOS_Range_D' 'LOS_Range_num_31to40' 'Q17_res_before_D' 'Q17_res_before_num_Own' 'Q18_res_after_D' 'Q18_res_after_num_Nursing' 'Q18_res_after_num_Own' 'Q23_30_Venlafaxine_num' 'Q30_16_Mirtazapine_num' 'Q30_30_Venlafaxine_num' 'Q32_1_Donepezil_num' 'Q37_16_Mirtazapine_num' 'Q37_25_Sertraline_num' 'Q37_30_Venlafaxine_num' 'drugsequence_A_5_8_presc_num' 'drugsequences5_8_3_10_6_11_7_9_presc_num' 'drugsequenceALLtotalexclpromethlith_presc_D' 'drugsequence_A_5_8_antidep_presc_num' 'drugsequence_A_5_8_dem_drugs_presc_num' 'drugsequences5_8_3_10_6_11_7_9_antidep_presc_num' 'drugsequences5_8_3_10_7_9_presc_num' 'drugsequences_antidep_5_8_3_10_7_9_presc_D' 'Antidepressants_reg_pres_presc_num' 'All_classes_2A_prescription_countpresc_den' 'All_classes_2B_prescription_countpresc_den' 'All_classes_2C_prescription_countpresc_den' 'Antidepressants2A_Count_presc_num' 'Antidepressants2B_Count_presc_num' 'DrugsDem2B_Count_presc_num' 'Antidepressants2C_Count_presc_num' 'DrugsDem2C_Count_presc_num' 'Antidepressant_review_presc_num' 'Dementia_med_review_rec_presc_num' 'Antidepressant_review_rec_presc_num'

**'Q23_30_Venlafaxine_num'**

'Q6_Prim_diagnosis_num_Cancer' 'Q7_no_Del_on_adm' 'Q8_no_Del_dur_adm' 'Q9_dem_subtype_num_Frontotemporal' 'Q9_dem_subtype_num_Vascular' 'Q23_16_Mirtazapine_num' 'Q30_30_Venlafaxine_num' 'Q37_30_Venlafaxine_num' 'drugsequences5_8_3_10_6_11_7_9_presc_num' 'drugsequence_A_5_8_antidep_presc_num' 'drugsequences5_8_3_10_6_11_7_9_antidep_presc_num' 'drugsequences5_8_3_10_7_9_presc_num' 'drugsequences_antidep_5_8_3_10_7_9_presc_D' 'Antidepressants_reg_pres_presc_num' 'All_classes_2A_prescription_countpresc_den' 'Antidepressants2A_Count_presc_num' 'Antidepressants2B_Count_presc_num' 'Antidepressants2C_Count_presc_num' 'Antidepressant_review_rec_presc_num'

**'Q29_2_Lamotrigine_num'**

'Q5_Ward_Recoded_num_Stroke' 'Q9_dem_subtype_num_Frontotemporal' 'Q22_2_Lamotrigine_num' 'Q36_2_Lamotrigine_num' 'Q37_10_Fluoxetine_num' 'drugsequence_A_5_8_anticonvulsants_presc_num' 'drugsequence_D_7_9_hypnotics_presc_num' 'drugsequences5_8_3_10_6_11_7_9_anticonvulsants_presc_num' 'drugsequences_anticon_5_8_3_10_7_9_presc_D' 'Anticonvulsants_reg_pres_presc_num' 'Anticon2A_Count_presc_num' 'Anticon2B_Count_presc_num' 'Anticon2C_Count_presc_num' 'Antidep_new_pres_symptoms_rec_sleep_disturbance_presc_num' 'Antidep_new_prescriptions_recby_ward_presc_num' 'Anticonvulsant_review_presc_num' 'Anticonvulsant_review_rec_presc_num' 'Antidep_new_prescriptions__reviewed_presc_num' 'Antidep_new_prescriptions_recommended_for_review_presc_num'

**'Q30_16_Mirtazapine_num'**

'N_BREAK' 'Q1_Age_Recoded_D' 'Q1_Age_Recoded_num_upto65' 'Q1_Age_Recoded_num_81to100' 'Q1_Age_min' 'Q2_Gender_D' 'Q2_Gender_num_female' 'Q3_Ethnicity_Recoded_D' 'Q4_Language_Recoded_D' 'Q5_Ward_Recoded_D' 'Q6_Prim_diagnosis_D' 'Q7_Del_on_adm_D' 'Q7_no_Del_on_adm' 'Q8_Del_dur_adm_D' 'Q8_no_Del_dur_adm' 'Q9_dem_subtype_D' 'Q9_dem_subtype_num_Frontotemporal' 'Q10_ICD_10' 'Q10_10_none_num' 'Q11_died_D' 'Q11_died_no_num' 'Q12_self_disc_D' 'Q12_self_disc_no_num' 'Q13_fast_track_D' 'Q13_fast_track_no_num' 'Q14_EOL_D' 'Q14_EOL_no_num' 'LOS_Range_D' 'Q17_res_before_D' 'Q18_res_after_D' 'Q23_16_Mirtazapine_num' 'Q32_1_Donepezil_num' 'Q37_16_Mirtazapine_num' 'Q37_30_Venlafaxine_num' 'drugsequence_A_5_8_presc_num' 'drugsequences5_8_3_10_6_11_7_9_presc_num' 'drugsequenceALLtotalexclpromethlith_presc_D' 'drugsequence_A_5_8_antidep_presc_num' 'drugsequence_A_5_8_dem_drugs_presc_num' 'drugsequences5_8_3_10_6_11_7_9_antidep_presc_num' 'drugsequences5_8_3_10_7_9_presc_num' 'drugsequences_antidep_5_8_3_10_7_9_presc_D' 'drugsequences_antidep_2_12_presc_num' 'Antidepressants_reg_pres_presc_num' 'All_classes_2A_prescription_countpresc_den' 'All_classes_2B_prescription_countpresc_den' 'All_classes_2C_prescription_countpresc_den' 'Antidepressants2A_Count_presc_num' 'Antidepressants2B_Count_presc_num' 'DrugsDem2B_Count_presc_num' 'Antidepressants2C_Count_presc_num' 'Antidepressant_review_presc_num' 'Antidepressant_review_rec_presc_num' 'Antidep_new_prescriptions__reviewed_presc_num'

**'Q30_30_Venlafaxine_num'**

'Q5_Ward_Recoded_num_Orthopaedics' 'Q6_Prim_diagnosis_num_Cancer' 'Q7_no_Del_on_adm' 'Q8_no_Del_dur_adm' 'Q9_dem_subtype_num_Frontotemporal' 'Q9_dem_subtype_num_Vascular' 'Q10_10_none_num' 'Q17_res_before_num_Nursing' 'Q23_16_Mirtazapine_num' 'Q23_30_Venlafaxine_num' 'Q30_4_Citalopram_num' 'Q37_4_Citalopram_num' 'Q37_30_Venlafaxine_num' 'drugsequence_A_5_8_presc_num' 'drugsequences5_8_3_10_6_11_7_9_presc_num' 'drugsequenceALLtotalexclpromethlith_presc_D' 'drugsequence_A_5_8_antidep_presc_num' 'drugsequences5_8_3_10_6_11_7_9_antidep_presc_num' 'drugsequences5_8_3_10_7_9_presc_num' 'drugsequences_antidep_5_8_3_10_7_9_presc_D' 'Antidepressants_reg_pres_presc_num' 'All_classes_2A_prescription_countpresc_den' 'All_classes_2B_prescription_countpresc_den' 'All_classes_2C_prescription_countpresc_den' 'Antidepressants2A_Count_presc_num' 'Antidepressants2B_Count_presc_num' 'Antidepressants2C_Count_presc_num' 'Antidepressant_review_rec_presc_num'

**'Q36_2_Lamotrigine_num'**

'Q5_Ward_Recoded_num_Stroke' 'Q9_dem_subtype_num_Frontotemporal' 'Q22_2_Lamotrigine_num' 'Q29_2_Lamotrigine_num' 'Q30_10_Fluoxetine_num' 'Q37_10_Fluoxetine_num' 'drugsequence_A_5_8_anticonvulsants_presc_num' 'drugsequence_B_3_10_anticonvulsants_presc_num' 'drugsequences5_8_3_10_6_11_7_9_anticonvulsants_presc_num' 'drugsequences_anticon_5_8_3_10_7_9_presc_D' 'Anticonvulsants_reg_pres_presc_num' 'Anticon2A_Count_presc_num' 'Anticon2C_Count_presc_num' 'Anticonvulsant_review_presc_num' 'Anticonvulsant_review_rec_presc_num' 'Antidep_new_prescriptions__reviewed_presc_num' 'Antidep_new_prescriptions_recommended_for_review_presc_num'

**'Q37_16_Mirtazapine_num'**

'N_BREAK' 'Q1_Age_Recoded_D' 'Q1_Age_Recoded_num_upto65' 'Q1_Age_Recoded_num_81to100' 'Q1_Age_min' 'Q2_Gender_D' 'Q2_Gender_num_female' 'Q3_Ethnicity_Recoded_D' 'Q3_Ethnicity_Recoded_num_white' 'Q4_Language_Recoded_D' 'Q5_Ward_Recoded_D' 'Q6_Prim_diagnosis_D' 'Q6_Prim_diagnosis_num_respiratory' 'Q7_Del_on_adm_D' 'Q7_no_Del_on_adm' 'Q8_Del_dur_adm_D' 'Q8_no_Del_dur_adm' 'Q9_dem_subtype_D' 'Q9_dem_subtype_num_Frontotemporal' 'Q10_ICD_10' 'Q10_10_none_num' 'Q11_died_D' 'Q11_died_no_num' 'Q12_self_disc_D' 'Q12_self_disc_no_num' 'Q13_fast_track_D' 'Q13_fast_track_no_num' 'Q14_EOL_D' 'Q14_EOL_no_num' 'LOS_Range_D' 'LOS_Range_num_31to40' 'Q17_res_before_D' 'Q17_res_before_num_Own' 'Q18_res_after_D' 'Q18_res_after_num_Nursing' 'Q23_16_Mirtazapine_num' 'Q30_16_Mirtazapine_num' 'Q32_1_Donepezil_num' 'drugsequence_A_5_8_presc_num' 'drugsequences5_8_3_10_6_11_7_9_presc_num' 'drugsequenceALLtotalexclpromethlith_presc_D' 'drugsequence_A_5_8_antidep_presc_num' 'drugsequence_A_5_8_dem_drugs_presc_num' 'drugsequences5_8_3_10_6_11_7_9_antidep_presc_num' 'drugsequences5_8_3_10_7_9_presc_num' 'drugsequences_antidep_5_8_3_10_7_9_presc_D' 'drugsequences_antidep_2_12_presc_num' 'drugsequences_antidep_2_12_4_presc_num' 'Antidepressants_reg_pres_presc_num' 'All_classes_2A_prescription_countpresc_den' 'All_classes_2B_prescription_countpresc_den' 'All_classes_2C_prescription_countpresc_den' 'Antidepressants2A_Count_presc_num' 'Antidepressants2B_Count_presc_num' 'DrugsDem2B_Count_presc_num' 'Antidepressants2C_Count_presc_num' 'DrugsDem2C_Count_presc_num' 'Antidep_new_pres_symptoms_rec_depression_presc_num' 'Antidep_new_prescriptions_recby_consultant_presc_num' 'Antidepressant_review_presc_num' 'Dementia_med_review_rec_presc_num' 'Antidepressant_review_rec_presc_num' 'Antidep_new_prescriptions__reviewed_presc_num'

**'Q37_27_Trazodone_num'**

'Q9_dem_subtype_num_Frontotemporal' 'Q18_res_after_num_longstay' 'S2A_Amisulpride_num' 'Q23_27_Trazodone_num' 'Q28_1_Amisulpride_num' 'Q30_27_Trazodone_num' 'Q35_1_Amisulpride_num' 'drugsequences_antidep_5_8_3_10_7_9_presc_D' 'drugsequences_antidep_2_12_presc_num' 'drugsequences_antidep_2_12_4_presc_num' 'Combined_new_presc_2_4_recom_for_review_presc_num' 'Combined_new_presc_2_4_rev_recom_presc_num' 'Antidepressants_reg_pres_presc_num' 'Antidepressants2B_Count_presc_num' 'Antidepressants2C_Count_presc_num' 'Demdrug_new_prescriptions_recby_consultant_presc_num' 'Antidepressant_review_rec_presc_num' 'Antidep_new_prescriptions__reviewed_presc_num' 'Antidep_new_prescriptions_recommended_for_review_presc_num'

**'Q37_30_Venlafaxine_num'**

'Q1_Age_Recoded_num_66to80' 'Q5_Ward_Recoded_num_Orthopaedics' 'Q7_no_Del_on_adm' 'Q8_no_Del_dur_adm' 'Q9_dem_subtype_num_Frontotemporal' 'Q9_dem_subtype_num_Vascular' 'Q10_10_none_num' 'Q23_16_Mirtazapine_num' 'Q23_30_Venlafaxine_num' 'Q30_16_Mirtazapine_num' 'Q30_30_Venlafaxine_num' 'drugsequence_A_5_8_presc_num' 'drugsequences5_8_3_10_6_11_7_9_presc_num' 'drugsequenceALLtotalexclpromethlith_presc_D' 'drugsequence_A_5_8_antidep_presc_num' 'drugsequences5_8_3_10_6_11_7_9_antidep_presc_num' 'drugsequences5_8_3_10_7_9_presc_num' 'drugsequences_antidep_5_8_3_10_7_9_presc_D' 'Antidepressants_reg_pres_presc_num' 'All_classes_2A_prescription_countpresc_den' 'All_classes_2B_prescription_countpresc_den' 'All_classes_2C_prescription_countpresc_den' 'Antidepressants2A_Count_presc_num' 'Antidepressants2B_Count_presc_num' 'Antidepressants2C_Count_presc_num' 'Antidepressant_review_rec_presc_num'

**'Q23_4_Citalopram_num'**

'N_BREAK' 'Q1_Age_Recoded_D' 'Q1_Age_Recoded_num_81to100' 'Q2_Gender_D' 'Q2_Gender_num_female' 'Q2_Gender_num_male' 'Q3_Ethnicity_Recoded_D' 'Q4_Language_Recoded_D' 'Q4_Language_Recoded_num_English' 'Q5_Ward_Recoded_D' 'Q6_Prim_diagnosis_D' 'Q7_Del_on_adm_D' 'Q7_no_Del_on_adm' 'Q8_Del_dur_adm_D' 'Q8_no_Del_dur_adm' 'Q9_dem_subtype_D' 'Q9_dem_subtype_num_Vascular' 'Q10_ICD_10' 'Q10_1_yes_bipolar_num' 'Q10_10_none_num' 'Q11_died_D' 'Q11_died_no_num' 'Q12_self_disc_D' 'Q12_self_disc_no_num' 'Q13_fast_track_D' 'Q13_fast_track_no_num' 'Q14_EOL_D' 'Q14_EOL_no_num' 'LOS_Range_D' 'LOS_Range_num_1to10' 'LOS_Range_num_11to20' 'Q17_res_before_D' 'Q17_res_before_num_Nursing' 'Q18_res_after_D' 'Q18_res_after_num_Nursing' 'Q22_4_Valproate_num' 'Q24_9_Diazepam_num' 'Q29_4_Valproate_num' 'Q30_4_Citalopram_num' 'Q37_4_Citalopram_num' 'drugsequence_A_5_8_presc_num' 'drugsequences5_8_3_10_6_11_7_9_presc_num' 'drugsequenceALLtotalexclpromethlith_presc_D' 'drugsequence_A_5_8_antidep_presc_num' 'drugsequence_C_6_11_antidep_presc_num' 'drugsequences5_8_3_10_6_11_7_9_hypnotics_presc_num' 'drugsequences5_8_3_10_6_11_7_9_antidep_presc_num' 'drugsequences5_8_3_10_7_9_presc_num' 'drugsequences_antidep_5_8_3_10_7_9_presc_D' 'drugsequences_hypnotics_5_8_3_10_7_9_presc_D' 'drugsequence_2_4_presc_num' 'Antidepressants_reg_pres_presc_num' 'All_classes_2A_prescription_countpresc_den' 'All_classes_2B_prescription_countpresc_den' 'All_classes_2C_prescription_countpresc_den' 'Antidepressants2A_Count_presc_num' 'Hypnotics2A_Count_presc_num' 'Antidepressants2B_Count_presc_num' 'Hypnotics2B_Count_presc_num' 'Antidepressants2C_Count_presc_num' 'Hypnotics2C_Count_presc_num' 'Antidepressant_review_presc_num' 'Antidepressant_review_rec_presc_num'

**'Q23_25_Sertraline_num'**

'N_BREAK' 'Q1_Age_Recoded_D' 'Q1_Age_Recoded_num_66to80' 'Q2_Gender_D' 'Q2_Gender_num_female' 'Q3_Ethnicity_Recoded_D' 'Q4_Language_Recoded_D' 'Q5_Ward_Recoded_D' 'Q5_Ward_Recoded_num_Surgical' 'Q6_Prim_diagnosis_D' 'Q7_Del_on_adm_D' 'Q8_Del_dur_adm_D' 'Q8_no_Del_dur_adm' 'Q9_dem_subtype_D' 'Q9_dem_subtype_num_Vascular' 'Q10_ICD_10' 'Q10_2_yes_depress_num' 'Q10_10_none_num' 'Q11_died_D' 'Q11_died_no_num' 'Q12_self_disc_D' 'Q12_self_disc_no_num' 'Q13_fast_track_D' 'Q13_fast_track_no_num' 'Q14_EOL_D' 'Q14_EOL_no_num' 'LOS_Range_D' 'LOS_Range_num_1to10' 'Q17_res_before_D' 'Q17_res_before_num_Nursing' 'Q18_res_after_D' 'Q24_9_Diazepam_num' 'Q30_25_Sertraline_num' 'Q31_9_Diazepam_num' 'Q37_25_Sertraline_num' 'Q38_9_Diazepam_num' 'drugsequence_A_5_8_presc_num' 'drugsequences5_8_3_10_6_11_7_9_presc_num' 'drugsequenceALLtotalexclpromethlith_presc_D' 'drugsequence_A_5_8_antidep_presc_num' 'drugsequences5_8_3_10_6_11_7_9_antidep_presc_num' 'drugsequences5_8_3_10_7_9_presc_num' 'drugsequences_antidep_5_8_3_10_7_9_presc_D' 'Antidepressants_reg_pres_presc_num' 'All_classes_2A_prescription_countpresc_den' 'All_classes_2B_prescription_countpresc_den' 'All_classes_2C_prescription_countpresc_den' 'Antidepressants2A_Count_presc_num' 'Antidepressants2B_Count_presc_num' 'Hypnotics2B_Count_presc_num' 'Antidepressants2C_Count_presc_num' 'Antidepressant_review_presc_num' 'Antidepressant_review_rec_presc_num'

**'Q23_30_Venlafaxine_num'**

'Q6_Prim_diagnosis_num_Cancer' 'Q7_no_Del_on_adm' 'Q8_no_Del_dur_adm' 'Q9_dem_subtype_num_Frontotemporal' 'Q9_dem_subtype_num_Vascular' 'Q23_16_Mirtazapine_num' 'Q30_30_Venlafaxine_num' 'Q37_30_Venlafaxine_num' 'drugsequences5_8_3_10_6_11_7_9_presc_num' 'drugsequence_A_5_8_antidep_presc_num' 'drugsequences5_8_3_10_6_11_7_9_antidep_presc_num' 'drugsequences5_8_3_10_7_9_presc_num' 'drugsequences_antidep_5_8_3_10_7_9_presc_D' 'Antidepressants_reg_pres_presc_num' 'All_classes_2A_prescription_countpresc_den' 'Antidepressants2A_Count_presc_num' 'Antidepressants2B_Count_presc_num' 'Antidepressants2C_Count_presc_num' 'Antidepressant_review_rec_presc_num'

**'Q30_2_Amitriptyline_num'**

'Q6_Prim_diagnosis_num_Endocrine' 'Q9_dem_subtype_num_Vascular' 'Q10_2_yes_depress_num' 'LOS_Range_num_1to10' 'S2A_Olanzapine_num' 'Q23_2_Amitriptyline_num' 'Q37_2_Amitriptyline_num' 'drugsequence_A_5_8_presc_num' 'drugsequences5_8_3_10_6_11_7_9_presc_num' 'drugsequenceALLtotalexclpromethlith_presc_D' 'drugsequence_A_5_8_antipsychotics_presc_num' 'drugsequences5_8_3_10_6_11_7_9_antipsychotics_presc_num' 'drugsequences5_8_3_10_6_11_7_9_hypnotics_presc_num' 'drugsequences5_8_3_10_6_11_7_9_antidep_presc_num' 'drugsequences5_8_3_10_7_9_presc_num' 'drugsequences_antidep_5_8_3_10_7_9_presc_D' 'drugsequences_hypnotics_5_8_3_10_7_9_presc_D' 'drugsequences_antipsych_5_8_3_10_7_9_presc_D' 'Antipsychotics_reg_pres_presc_num' 'Antidepressants_reg_pres_presc_num' 'All_classes_2A_prescription_countpresc_den' 'All_classes_2B_prescription_countpresc_den' 'All_classes_2C_prescription_countpresc_den' 'Antipsychotics2A_Count_presc_num' 'Antidepressants2A_Count_presc_num' 'Hypnotics2A_Count_presc_num' 'Antipsychotics2B_Count_presc_num' 'Antidepressants2B_Count_presc_num' 'Hypnotics2B_Count_presc_num' 'Antipsychotics2C_Count_presc_num' 'Hypnotics2C_Count_presc_num' 'Antipsych_new_prescriptions_recby_out_of_hours_presc_num' 'Antipsychotics_review_presc_num' 'Antipsychotics_review_rec_presc_num'

**'Q30_4_Citalopram_num'**

'N_BREAK' 'Q1_Age_Recoded_D' 'Q1_Age_Recoded_num_81to100' 'Q2_Gender_D' 'Q2_Gender_num_male' 'Q3_Ethnicity_Recoded_D' 'Q4_Language_Recoded_D' 'Q4_Language_Recoded_num_English' 'Q5_Ward_Recoded_D' 'Q6_Prim_diagnosis_D' 'Q7_Del_on_adm_D' 'Q7_no_Del_on_adm' 'Q8_Del_dur_adm_D' 'Q8_no_Del_dur_adm' 'Q9_dem_subtype_D' 'Q9_dem_subtype_num_Vascular' 'Q10_ICD_10' 'Q10_10_none_num' 'Q11_died_D' 'Q11_died_no_num' 'Q12_self_disc_D' 'Q12_self_disc_no_num' 'Q13_fast_track_D' 'Q13_fast_track_no_num' 'Q14_EOL_D' 'Q14_EOL_no_num' 'LOS_Range_D' 'LOS_Range_num_1to10' 'LOS_Range_num_11to20' 'Q17_res_before_D' 'Q17_res_before_num_Nursing' 'Q18_res_after_D' 'Q18_res_after_num_Nursing' 'Q23_4_Citalopram_num' 'Q24_9_Diazepam_num' 'Q30_30_Venlafaxine_num' 'Q31_9_Diazepam_num' 'Q37_4_Citalopram_num' 'Q38_9_Diazepam_num' 'drugsequence_A_5_8_presc_num' 'drugsequences5_8_3_10_6_11_7_9_presc_num' 'drugsequenceALLtotalexclpromethlith_presc_D' 'drugsequence_A_5_8_antidep_presc_num' 'drugsequence_C_6_11_antidep_presc_num' 'drugsequences5_8_3_10_6_11_7_9_hypnotics_presc_num' 'drugsequences5_8_3_10_6_11_7_9_antidep_presc_num' 'drugsequences5_8_3_10_7_9_presc_num' 'drugsequences_antidep_5_8_3_10_7_9_presc_D' 'drugsequences_hypnotics_5_8_3_10_7_9_presc_D' 'Antidepressants_reg_pres_presc_num' 'All_classes_2A_prescription_countpresc_den' 'All_classes_2B_prescription_countpresc_den' 'All_classes_2C_prescription_countpresc_den' 'Antidepressants2A_Count_presc_num' 'Hypnotics2A_Count_presc_num' 'Antidepressants2B_Count_presc_num' 'Hypnotics2B_Count_presc_num' 'Antidepressants2C_Count_presc_num' 'Hypnotics2C_Count_presc_num' 'Antidepressant_review_presc_num' 'Antidepressant_review_rec_presc_num'

**'Q30_25_Sertraline_num'**

'N_BREAK' 'Q1_Age_Recoded_D' 'Q1_Age_Recoded_num_66to80' 'Q2_Gender_D' 'Q2_Gender_num_female' 'Q3_Ethnicity_Recoded_D' 'Q4_Language_Recoded_D' 'Q5_Ward_Recoded_D' 'Q5_Ward_Recoded_num_Other' 'Q6_Prim_diagnosis_D' 'Q7_Del_on_adm_D' 'Q8_Del_dur_adm_D' 'Q9_dem_subtype_D' 'Q9_dem_subtype_num_Vascular' 'Q10_ICD_10' 'Q10_2_yes_depress_num' 'Q10_10_none_num' 'Q11_died_D' 'Q11_died_no_num' 'Q12_self_disc_D' 'Q12_self_disc_no_num' 'Q13_fast_track_D' 'Q13_fast_track_no_num' 'Q14_EOL_D' 'Q14_EOL_no_num' 'LOS_Range_D' 'LOS_Range_num_1to10' 'Q17_res_before_D' 'Q17_res_before_num_Nursing' 'Q18_res_after_D' 'Q23_25_Sertraline_num' 'Q24_9_Diazepam_num' 'Q31_9_Diazepam_num' 'Q37_25_Sertraline_num' 'drugsequence_A_5_8_presc_num' 'drugsequences5_8_3_10_6_11_7_9_presc_num' 'drugsequenceALLtotalexclpromethlith_presc_D' 'drugsequence_A_5_8_antidep_presc_num' 'drugsequences5_8_3_10_6_11_7_9_antidep_presc_num' 'drugsequences5_8_3_10_7_9_presc_num' 'drugsequences_antidep_5_8_3_10_7_9_presc_D' 'Antidepressants_reg_pres_presc_num' 'All_classes_2A_prescription_countpresc_den' 'All_classes_2B_prescription_countpresc_den' 'All_classes_2C_prescription_countpresc_den' 'Antidepressants2A_Count_presc_num' 'Antidepressants2B_Count_presc_num' 'Hypnotics2B_Count_presc_num' 'Antidepressants2C_Count_presc_num' 'Hypnotics_new_prescriptions_recby_consultant_presc_num' 'Antidepressant_review_presc_num' 'Antidepressant_review_rec_presc_num'

**'Q30_30_Venlafaxine_num'**

'Q5_Ward_Recoded_num_Orthopaedics' 'Q6_Prim_diagnosis_num_Cancer' 'Q7_no_Del_on_adm' 'Q8_no_Del_dur_adm' 'Q9_dem_subtype_num_Frontotemporal' 'Q9_dem_subtype_num_Vascular' 'Q10_10_none_num' 'Q17_res_before_num_Nursing' 'Q23_16_Mirtazapine_num' 'Q23_30_Venlafaxine_num' 'Q30_4_Citalopram_num' 'Q37_4_Citalopram_num' 'Q37_30_Venlafaxine_num' 'drugsequence_A_5_8_presc_num' 'drugsequences5_8_3_10_6_11_7_9_presc_num' 'drugsequenceALLtotalexclpromethlith_presc_D' 'drugsequence_A_5_8_antidep_presc_num' 'drugsequences5_8_3_10_6_11_7_9_antidep_presc_num' 'drugsequences5_8_3_10_7_9_presc_num' 'drugsequences_antidep_5_8_3_10_7_9_presc_D' 'Antidepressants_reg_pres_presc_num' 'All_classes_2A_prescription_countpresc_den' 'All_classes_2B_prescription_countpresc_den' 'All_classes_2C_prescription_countpresc_den' 'Antidepressants2A_Count_presc_num' 'Antidepressants2B_Count_presc_num' 'Antidepressants2C_Count_presc_num' 'Antidepressant_review_rec_presc_num'

**'Q37_4_Citalopram_num'**

'N_BREAK' 'Q1_Age_Recoded_D' 'Q1_Age_Recoded_num_66to80' 'Q1_Age_Recoded_num_81to100' 'Q2_Gender_D' 'Q2_Gender_num_male' 'Q3_Ethnicity_Recoded_D' 'Q4_Language_Recoded_D' 'Q4_Language_Recoded_num_English' 'Q5_Ward_Recoded_D' 'Q6_Prim_diagnosis_D' 'Q7_Del_on_adm_D' 'Q7_no_Del_on_adm' 'Q8_Del_dur_adm_D' 'Q8_no_Del_dur_adm' 'Q9_dem_subtype_D' 'Q9_dem_subtype_num_Vascular' 'Q10_ICD_10' 'Q10_10_none_num' 'Q11_died_D' 'Q11_died_no_num' 'Q12_self_disc_D' 'Q12_self_disc_no_num' 'Q13_fast_track_D' 'Q13_fast_track_no_num' 'Q14_EOL_D' 'Q14_EOL_no_num' 'LOS_Range_D' 'LOS_Range_num_1to10' 'LOS_Range_num_11to20' 'Q17_res_before_D' 'Q17_res_before_num_Nursing' 'Q17_res_before_num_Own' 'Q18_res_after_D' 'Q18_res_after_num_Nursing' 'Q23_4_Citalopram_num' 'Q24_9_Diazepam_num' 'Q30_4_Citalopram_num' 'Q30_30_Venlafaxine_num' 'Q31_9_Diazepam_num' 'Q38_9_Diazepam_num' 'drugsequence_A_5_8_presc_num' 'drugsequences5_8_3_10_6_11_7_9_presc_num' 'drugsequenceALLtotalexclpromethlith_presc_D' 'drugsequence_A_5_8_antidep_presc_num' 'drugsequences5_8_3_10_6_11_7_9_hypnotics_presc_num' 'drugsequences5_8_3_10_6_11_7_9_antidep_presc_num' 'drugsequences5_8_3_10_7_9_presc_num' 'drugsequences_antidep_5_8_3_10_7_9_presc_D' 'drugsequences_hypnotics_5_8_3_10_7_9_presc_D' 'Antidepressants_reg_pres_presc_num' 'All_classes_2A_prescription_countpresc_den' 'All_classes_2B_prescription_countpresc_den' 'All_classes_2C_prescription_countpresc_den' 'Antidepressants2A_Count_presc_num' 'Hypnotics2A_Count_presc_num' 'Antidepressants2B_Count_presc_num' 'Antidepressants2C_Count_presc_num' 'Hypnotics2C_Count_presc_num' 'Antidepressant_review_presc_num' 'Antidepressant_review_rec_presc_num'

**'Q37_25_Sertraline_num'**

'N_BREAK' 'Q1_Age_Recoded_D' 'Q1_Age_Recoded_num_66to80' 'Q1_Age_Recoded_num_81to100' 'Q2_Gender_D' 'Q2_Gender_num_female' 'Q3_Ethnicity_Recoded_D' 'Q4_Language_Recoded_D' 'Q5_Ward_Recoded_D' 'Q5_Ward_Recoded_num_Other' 'Q6_Prim_diagnosis_D' 'Q7_Del_on_adm_D' 'Q7_no_Del_on_adm' 'Q8_Del_dur_adm_D' 'Q8_no_Del_dur_adm' 'Q9_dem_subtype_D' 'Q9_dem_subtype_num_Vascular' 'Q10_ICD_10' 'Q10_2_yes_depress_num' 'Q10_10_none_num' 'Q11_died_D' 'Q11_died_no_num' 'Q12_self_disc_D' 'Q12_self_disc_no_num' 'Q13_fast_track_D' 'Q13_fast_track_no_num' 'Q14_EOL_D' 'Q14_EOL_no_num' 'LOS_Range_D' 'LOS_Range_num_1to10' 'Q17_res_before_D' 'Q17_res_before_num_Nursing' 'Q18_res_after_D' 'Q18_res_after_num_Nursing' 'Q23_16_Mirtazapine_num' 'Q23_25_Sertraline_num' 'Q24_9_Diazepam_num' 'Q30_25_Sertraline_num' 'Q31_9_Diazepam_num' 'Q38_9_Diazepam_num' 'drugsequence_A_5_8_presc_num' 'drugsequences5_8_3_10_6_11_7_9_presc_num' 'drugsequenceALLtotalexclpromethlith_presc_D' 'drugsequence_A_5_8_antidep_presc_num' 'drugsequences5_8_3_10_6_11_7_9_antidep_presc_num' 'drugsequences5_8_3_10_7_9_presc_num' 'drugsequences_antidep_5_8_3_10_7_9_presc_D' 'drugsequences_hypnotics_5_8_3_10_7_9_presc_D' 'Antidepressants_reg_pres_presc_num' 'All_classes_2A_prescription_countpresc_den' 'All_classes_2B_prescription_countpresc_den' 'All_classes_2C_prescription_countpresc_den' 'Antidepressants2A_Count_presc_num' 'Antidepressants2B_Count_presc_num' 'Hypnotics2B_Count_presc_num' 'Antidepressants2C_Count_presc_num' 'Hypnotics_new_prescriptions_recby_consultant_presc_num' 'Antidepressant_review_presc_num' 'Antidepressant_review_rec_presc_num'

**'Q37_30_Venlafaxine_num'**

'Q1_Age_Recoded_num_66to80' 'Q5_Ward_Recoded_num_Orthopaedics' 'Q7_no_Del_on_adm' 'Q8_no_Del_dur_adm' 'Q9_dem_subtype_num_Frontotemporal' 'Q9_dem_subtype_num_Vascular' 'Q10_10_none_num' 'Q23_16_Mirtazapine_num' 'Q23_30_Venlafaxine_num' 'Q30_16_Mirtazapine_num' 'Q30_30_Venlafaxine_num' 'drugsequence_A_5_8_presc_num' 'drugsequences5_8_3_10_6_11_7_9_presc_num' 'drugsequenceALLtotalexclpromethlith_presc_D' 'drugsequence_A_5_8_antidep_presc_num' 'drugsequences5_8_3_10_6_11_7_9_antidep_presc_num' 'drugsequences5_8_3_10_7_9_presc_num' 'drugsequences_antidep_5_8_3_10_7_9_presc_D' 'Antidepressants_reg_pres_presc_num' 'All_classes_2A_prescription_countpresc_den' 'All_classes_2B_prescription_countpresc_den' 'All_classes_2C_prescription_countpresc_den' 'Antidepressants2A_Count_presc_num' 'Antidepressants2B_Count_presc_num' 'Antidepressants2C_Count_presc_num' 'Antidepressant_review_rec_presc_num'
