# Supplementary material for: Multi-dimensional relationships among dementia, depression and prescribed drugs in England and Wales hospitals
Source: BMC Med Inform Decis Mak. 2022 Oct 7;22:262. doi: 10.1186/s12911-022-01892-9 (PMC9547465; doi:10.1186/s12911-022-01892-9)
Supplement: Supplementary file 6 — Additional file 6. Dataset details and supplementary results that explore the relationships among dementia, depression, and prescribed drugs. [file 12911_2022_1892_MOESM6_ESM.docx]

**Multi-dimensional relationships among dementia, depression and prescribed drugs in England and Wales hospitals**

Alok Joshi^1,2*^, Stephen Todd^3^, David P. Finn^4^, Paula L. McClean^5^ and KongFatt Wong-Lin^1*^

^1^ Intelligent Systems Research Centre, Ulster University, Derry~Londonderry, Northern Ireland, UK

^2^ Department of Computer Science, University of Bath, Bath, UK

^3^ Altnagelvin Area Hospital, Western Health and Social Care Trust, Derry~Londonderry, Northern Ireland, UK

^4^ Pharmacology and Therapeutics, School of Medicine, Galway Neuroscience Centre, National University of Ireland Galway, Ireland

^5^ Northern Ireland Centre for Stratified Medicine, Biomedical Sciences Research Institute, Ulster University, Magee Campus, Derry~Londonderry, Northern Ireland, UK

^*^Corresponding authors: [a.joshi@ulster.ac.uk](mailto:a.joshi@ulster.ac.uk) ; [k.wong-lin@ulster.ac.uk](mailto:k.wong-lin@ulster.ac.uk)

This PDF file includes-

Dataset details, Supplementary Results: Table S1-S18.

**Supplementary section-**

**Dataset details-**

Ft_1_: total number of patient participated in the audit; Ft_2_ to Ft_10_: age related features; Ft_11_ to Ft_15_: gender specific features; Ft_16_ to Ft_22_: Ethnicity features; Ft_23_ to Ft_27_: language features; Ft_28_ to Ft_36_: patients with specific ward/team; Ft_37_ to Ft_62_: patients with primary diagnosis; Ft_63_ to Ft_68_: patients with delirium as a part of admitting condition; Ft_69_ to Ft_79_: patients with recorded dementia subtypes; Ft_80_ to Ft_90_: patients with psychiatric diagnosis; Ft_91_ to Ft_93_: patients died in the hospitals; Ft_94_ to Ft_99_: patients details related to discharge from the hospital; Ft_100_ to Ft_102_: patients receiving end of life care or care plan; Ft_103_ to Ft_116_: patients with length of stay recorded; Ft_117_ to Ft_129_: place of residence recorded before admission; Ft_130_ to Ft_142_: place of residence recorded after discharge; Ft_143_ to Ft_196_: total number of prescriptions of specific drugs at the time of admission; Ft_197_ to Ft_249_: total number of prescriptions of specific drugs in the hospital; Ft_250_ to Ft_302_: total number of prescriptions of specific drugs at the time of discharge; Ft_303_ to Ft_311_: number of prescriptions under different scenarios; Ft_312_ to Ft_338_: total number of prescriptions related to antipsychotic, hypnotics, antidepressant, dementia and anticonvulsants; Ft_339_ to Ft_345_: total number of new prescriptions related to antipsychotic, hypnotics, antidepressant, dementia and anticonvulsants during different time in hospital; Ft_346_ to Ft_350_: number of new prescriptions related to antipsychotic, hypnotics, antidepressant, dementia and anticonvulsants; Ft_351_ to Ft_355_: total new prescriptions related to antipsychotic, hypnotics, antidepressant, dementia and anticonvulsants; Ft_356_ to Ft_357_: new prescriptions made during hospital/discharge or combinations; Ft_358_ to Ft_372_: total new prescriptions related to antipsychotic, hypnotics, antidepressant, dementia and anticonvulsants recommended for review post-discharge; Ft_373_ to Ft_380_: regular new prescriptions of antipsychotic, hypnotics, antidepressant, dementia and anticonvulsants; Ft_381_ to Ft_383_: total prescriptions on admission/ in hospital/ or during discharge; Ft_384_ to Ft_398_: total number of prescription of antipsychotic, hypnotics, antidepressant, dementia and anticonvulsants on admission/hospital/discharge; Ft_399_ to Ft_439_: antipsychotic, hypnotics, antidepressant, dementia and anticonvulsants prescription for reasons; Ft_440_ to Ft_480_: new prescriptions of antipsychotic, hypnotics, antidepressant, dementia and anticonvulsants reasons for prescription; Ft_481_ to Ft_510_: prescriptions recommended for review or reviewed at different times; Ft_511_ to Ft_530_: prescriptions recommended for review or reviewed at different times.

**Table S1: Prediction of number of Donepezil prescriptions based on number of AD patients.**

**Regression Summary:**

| Regression key values/Time | Admission | Hospital | Discharge |
| --- | --- | --- | --- |
| Regression equation | -0.0 + (0.6643*Q9_dem_subtype_num_Alzheimers) | 0.0 + (0.6746*Q9_dem_subtype_num_Alzheimers) | 0.0 + (0.6339*Q9_dem_subtype_num_Alzheimers) |
| Coefficient of determination  (r-squared) | 0.4413 | 0.4551 | 0.4018 |
| Adjusted  r-squared | 0.4297 | 0.4437 | 0.3893 |
| Root Mean Square Error (RMSE) | 0.7399 | 0.7308 | 0.7657 |
| Mean of Y | -0.0 | 0.0 | 0.0 |
| Residual standard error | 0.7552 | 0.7458 | 0.7815 |
| No. of Observations | 50 | 50 | 50 |

**Regression Coefficients:**

| Regression coefficients/  Time | Admission | | | | Hospital | | | | Discharge | | | |
| --- | --- | --- | --- | --- | --- | --- | --- | --- | --- | --- | --- | --- |
| Parameter | **Est** | **Std Err** | **T-val** | **P-val** | **Est** | **Std Err** | **T-val** | **P-val** | **Est** | **Std Err** | **T-val** | **P-val** |
| Intercept | -2.46855e-10 | 0.1068 | -2.31137e-09 | 1 | 2.9397e-10 | 0.1055 | 2.78644e-09 | 1 | 9.07083e-11 | 0.1105 | 8.2089e-10 | 1 |
| Q9_dem_sub  type_num_ Alzheimers | 0.664315 | 0.1079 | 6.15677 | 1.444e-07 | 0.674624 | 0.1065 | 6.3345 | 7.7153e-08 | 0.633854 | 0.1116 | 5.6797 | 7.7057e-07 |

**ANOVA Summary:**

|  | Admission | | | |  | Hospital | | | |  |  | | Discharge | | | |
| --- | --- | --- | --- | --- | --- | --- | --- | --- | --- | --- | --- | --- | --- | --- | --- | --- |
| Source | **Df** | **Sum**  **squares** | **Mean Squares** | **F** | **Pr(>F)** | **Df** | **Sum**  **squares** | **Mean Squares** | **F** | **Pr(>F)** | **Df** | **Sum**  **squares** | | **Mean Squares** | **F** | **Pr(>F)** |
| Model | 1 | 21.6244 | 21.6244 | 37.916 | 1.4398E-07 | 1 | 22.3008 | 22.3008 | 40.0924 | 7.7876E-08 | 1 | 19.6868 | | 19.6868 | 32.2368 | 7.7583E-07 |
| Error | 48 | 27.3756 | 0.5703 |  |  | 48 | 26.6992 | 0.5562 |  |  | 48 | 21.3132 | | 0.6107 |  |  |
| Total | 49 | 49 |  |  |  | 49 |  |  |  |  | 49 | 49 | |  |  |  |

**Table S2: Prediction of number of Donepezil prescriptions based on number of previous donepezil and mirtazapine prescriptions.**

**Regression Summary:**

| Regression key values/Time | Admission | Hospital | Discharge |
| --- | --- | --- | --- |
| Regression equation | - | 0.0 + (-0.2555*Q23_16_Mirtazapine_num) + (0.9186*Q25_1_Donepezil_num) + (0.3902*Q30_16_Mirtazapine_num) | - |
| Coefficient of determination  (r-squared) | - | 0.9237 | - |
| Adjusted  r-squared | - | 0.9187 | - |
| Root Mean Square Error (RMSE) | - | 0.2734 | - |
| Mean of Y | - | 0.0 | - |
| Residual standard error | - | 0.2851 | - |
| No. of Observations | - | 50 | - |

**Regression Coefficients:**

| Regression coefficients/  Time | Admission | | | | Hospital | | | | Discharge | | | |
| --- | --- | --- | --- | --- | --- | --- | --- | --- | --- | --- | --- | --- |
| Parameter | **Est** | **Std Err** | **T-val** | **P-val** | **Est** | **Std Err** | **T-val** | **P-val** | **Est** | **Std Err** | **T-val** | **P-val** |
| Intercept | - | - | - | - | 5.36027e-10 | 0.0403 | 1.33009e-08 | 1 | - | - | - | - |
| Q23_16_Mirtazapine_num | - | - | - | - | -0.255473 | 0.1158 | -2.20616 | 0.032406 | - | - | - | - |
| Q25_1_Donepezil_num | - | - | - | - | 0.918632 | 0.0437 | 21.0213 | 3.1672e-25 | - | - | - | - |
| Q30_16_Mirtazapine_num | - | - | - | - | 0.390237 | 0.1135 | 3.43821 | 0.0012538 | - | - | - | - |

**ANOVA Summary:**

|  | Admission | | | |  | Hospital | | | |  |  | | Discharge | | | |
| --- | --- | --- | --- | --- | --- | --- | --- | --- | --- | --- | --- | --- | --- | --- | --- | --- |
| Source | **Df** | **Sum**  **squares** | **Mean Squares** | **F** | **Pr(>F)** | **Df** | **Sum**  **squares** | **Mean Squares** | **F** | **Pr(>F)** | **Df** | **Sum**  **squares** | | **Mean Squares** | **F** | **Pr(>F)** |
| Model | - | - | - | - | - | 3 | 45.2604 | 15.0868 | 185.5792 | 1.0575E-25 | - | - | | - | - | - |
| Error | - | - | - |  |  | 46 | 3.7396 | 0.0813 |  |  | - | - | | - |  |  |
| Total | - | - |  |  |  | 49 | 49 |  |  |  | - | - | |  |  |  |

**Table S3: Prediction of number of Memantine prescriptions based on number of AD patients.**

**Regression Summary:**

| Regression key values/Time | Admission | Hospital | Discharge |
| --- | --- | --- | --- |
| Regression equation | 0.0 + (0.5782*Q9_dem_subtype_num_Alzheimers) | 0.0 + (0.6049*Q9_dem_subtype_num_Alzheimers) | 0.0 + (0.6235*Q9_dem_subtype_num_Alzheimers) |
| Coefficient of determination  (r-squared) | 0.3344 | 0.3659 | 0.3887 |
| Adjusted  r-squared | 0.3205 | 0.3527 | 0.376 |
| Root Mean Square Error (RMSE) | 0.8076 | 0.7883 | 0.774 |
| Mean of Y | 0.0 | 0.0 | 0.0 |
| Residual standard error | 0.8243 | 0.8045 | 0.7899 |
| No. of Observations | 50 | 50 | 50 |

**Regression Coefficients:**

| Regression coefficients/  Time | Admission | | | | Hospital | | | | Discharge | | | |
| --- | --- | --- | --- | --- | --- | --- | --- | --- | --- | --- | --- | --- |
| Parameter | **Est** | **Std Err** | **T-val** | **P-val** | **Est** | **Std Err** | **T-val** | **P-val** | **Est** | **Std Err** | **T-val** | **P-val** |
| Intercept | 1.06259e-10 | 0.1166 | 9.11316e-10 | 1 | 8.83934e-11 | 0.1138 | 7.76744e-10 | 1 | 8.98788e-11 | 0.1117 | 8.04644e-10 | 1 |
| Q9_dem_sub  type_num_ Alzheimers | 0.578243 | 0.1178 | 4.90869 | 1.0995e-05 | 0.604918 | 0.1149 | 5.26474 | 3.2539e-06 | 0.623485 | 0.1128 | 5.52735 | 1.3105e-06 |

**ANOVA Summary:**

|  | Admission | | | |  | Hospital | | | |  |  | | Discharge | | | |
| --- | --- | --- | --- | --- | --- | --- | --- | --- | --- | --- | --- | --- | --- | --- | --- | --- |
| Source | **Df** | **Sum**  **squares** | **Mean Squares** | **F** | **Pr(>F)** | **Df** | **Sum**  **squares** | **Mean Squares** | **F** | **Pr(>F)** | **Df** | **Sum**  **squares** | | **Mean Squares** | **F** | **Pr(>F)** |
| Model | 1 | 16.3839 | 16.3839 | 24.1116 | 1.0933E-05 | 1 | 17.9304 | 17.9304 | 27.701 | 3.2715E-06 | 1 | 19.0479 | | 19.0479 | 30.5255 | 1.3213E-06 |
| Error | 48 | 32.6161 | 0.6795 |  |  | 48 | 31.0696 | 0.6473 |  |  | 48 | 29.9521 | | 0.624 |  |  |
| Total | 49 |  |  |  |  | 49 |  |  |  |  | 49 |  | |  |  |  |

**Table S4: Prediction of number of Memantine prescriptions based on number of memantine and risperidone prescriptions.**

**Regression Summary:**

| Regression key values/Time | Admission | Hospital | Discharge |
| --- | --- | --- | --- |
| Regression equation | - | -0.0 + (0.9195*Q25_3_Memantine_num) + (0.119*Q28_23_Risperidone_num) | - |
| Coefficient of determination  (r-squared) | - | 0.9469 | - |
| Adjusted  r-squared | - | 0.9446 | - |
| Root Mean Square Error (RMSE) | - | 0.2281 | - |
| Mean of Y | - | 0.0 | - |
| Residual standard error | - | 0.2354 | - |
| No. of Observations | - | 50 | - |

**Regression Coefficients:**

| Regression coefficients/  Time | Admission | | | | Hospital | | | | Discharge | | | |
| --- | --- | --- | --- | --- | --- | --- | --- | --- | --- | --- | --- | --- |
| Parameter | **Est** | **Std Err** | **T-val** | **P-val** | **Est** | **Std Err** | **T-val** | **P-val** | **Est** | **Std Err** | **T-val** | **P-val** |
| Intercept | - | - | - | - | -1.99288e-11 | 0.0333 | -5.98464e-10 | 1 | - | - | - | - |
| Q25_3_Memantine_num | - | - | - | - | 0.919471 | 0.0367 | 25.0537 | 7.7442e-29 | - | - | - | - |
| Q28_23_Risperidone_num | - | - | - | - | 0.119015 | 0.0367 | 3.24291 | 0.0021797 | - | - | - | - |

**ANOVA Summary:**

|  | Admission | | | |  | Hospital | | | |  |  | | Discharge | | | |
| --- | --- | --- | --- | --- | --- | --- | --- | --- | --- | --- | --- | --- | --- | --- | --- | --- |
| Source | **Df** | **Sum**  **squares** | **Mean Squares** | **F** | **Pr(>F)** | **Df** | **Sum**  **squares** | **Mean Squares** | **F** | **Pr(>F)** | **Df** | **Sum**  **squares** | | **Mean Squares** | **F** | **Pr(>F)** |
| Model | - | - | - | - | - | 2 | 46.3963 | 23.1982 | 418.7575 | 1.1136E-30 | - | - | | - | - | - |
| Error | - | - | - |  |  | 47 | 2.60369 | 0.0554 |  |  | - | - | | - |  |  |
| Total | - | - |  |  |  | 49 | 49 |  |  |  | - | - | |  |  |  |

**Table S5: Prediction of number of Sertraline prescriptions based on number of VaD and depressed patients.**

**Regression Summary:**

| Regression key values/Time | Admission | Hospital | Discharge |
| --- | --- | --- | --- |
| Regression equation | -0.0 + (0.3046*Q9_dem_subtype_num_Vascular) + (0.335*Q10_2_yes_depress_num) | -0.0 + (0.2941*Q9_dem_subtype_num_Vascular) + (0.352*Q10_2_yes_depress_num) | -0.0 + (0.3169*Q9_dem_subtype_num_Vascular) + (0.3369*Q10_2_yes_depress_num) |
| Coefficient of determination  (r-squared) | 0.2844 | 0.291 | 0.2971 |
| Adjusted  r-squared | 0.2539 | 0.2608 | 0.2672 |
| Root Mean Square Error (RMSE) | 0.8374 | 0.8336 | 0.83 |
| Mean of Y | -0.0 | -0.0 | -0.0 |
| Residual standard error | 0.8638 | 0.8598 | 0.8561 |
| No. of Observations | 50 | 50 | 50 |

**Regression Coefficients:**

| Regression coefficients/  Time | Admission | | | | Hospital | | | | Discharge | | | |
| --- | --- | --- | --- | --- | --- | --- | --- | --- | --- | --- | --- | --- |
| Parameter | **Est** | **Std Err** | **T-val** | **P-val** | **Est** | **Std Err** | **T-val** | **P-val** | **Est** | **Std Err** | **T-val** | **P-val** |
| Intercept | -3.38101e-11 | 0.1222 | -2.76678e-10 | 1 | -1.29983e-11 | 0.1216 | -1.06894e-10 | 1 | -3.34452e-11 | 0.1211 | -2.76179e-10 | 1 |
| Q9_VaD | 0.304637 | 0.134 | 2.27341 | 0.027613 | 0.294136 | 0.1333 | 2.20657 | 0.032266 | 0.31691 | 0.1328 | 2.38637 | 0.021094 |
| Q10_2_depress | 0.334953 | 0.134 | 2.49965 | 0.015981 | 0.351983 | 0.1333 | 2.64053 | 0.0112 | 0.336943 | 0.1328 | 2.53722 | 0.014551 |

**ANOVA Summary:**

|  | Admission | | | |  | Hospital | | | |  |  | | Discharge | | | |
| --- | --- | --- | --- | --- | --- | --- | --- | --- | --- | --- | --- | --- | --- | --- | --- | --- |
| Source | **Df** | **Sum**  **squares** | **Mean Squares** | **F** | **Pr(>F)** | **Df** | **Sum**  **squares** | **Mean Squares** | **F** | **Pr(>F)** | **Df** | **Sum**  **squares** | | **Mean Squares** | **F** | **Pr(>F)** |
| Model | 2 | 13.9356 | 6.9678 | 9.3395 | 3.8438E-04 | 2 | 14.2575 | 7.1288 | 9.6439 | 3.0947E-04 | 2 | 14.5556 | | 7.2778 | 9.9307 | 2.5274E-04 |
| Error | 47 | 35.0644 | 0.7461 |  |  | 47 | 34.7425 | 0.7392 |  |  | 47 | 34.4444 | | 0.7329 |  |  |
| Total | 49 | 49 |  |  |  | 49 | 49 |  |  |  | 49 | 49 | |  |  |  |

**Table S6: Prediction of number of Sertraline prescriptions based on number of VaD and diazepam prescription.**

**Regression Summary:**

| Regression key values/Time | Admission | Hospital | Discharge |
| --- | --- | --- | --- |
| Regression equation | -0.0 + (0.3316*Q9_dem_subtype_num_Vascular) + (0.3239*Q24_9_Diazepam_num) | -0.0 + (0.3026*Q9_dem_subtype_num_Vascular) + (0.3576*Q31_9_Diazepam_num) | -0.0 + (0.3338*Q9_dem_subtype_num_Vascular) + (0.3219*Q38_9_Diazepam_num) |
| Coefficient of determination  (r-squared) | 0.2834 | 0.2972 | 0.2913 |
| Adjusted  r-squared | 0.2529 | 0.2673 | 0.2611 |
| Root Mean Square Error (RMSE) | 0.838 | 0.8299 | 0.8334 |
| Mean of Y | -0.0 | -0.0 | -0.0 |
| Residual standard error | 0.8643 | 0.856 | 0.8596 |
| No. of Observations | 50 | 50 | 50 |

**Regression Coefficients:**

| Regression coefficients/  Time | Admission | | | | Hospital | | | | Discharge | | | |
| --- | --- | --- | --- | --- | --- | --- | --- | --- | --- | --- | --- | --- |
| Parameter | **Est** | **Std Err** | **T-val** | **P-val** | **Est** | **Std Err** | **T-val** | **P-val** | **Est** | **Std Err** | **T-val** | **P-val** |
| Intercept | -5.33674e-11 | 0.1222 | -4.36722e-10 | 1 | -1.24914e-11 | 0.1211 | -1.0315e-10 | 1 | -1.56323e-10 | 0.1216 | -1.28555e-09 | 1 |
| Q9_VaD | 0.331631 | 0.1303 | 2.54514 | 0.014265 | 0.302635 | 0.131 | 2.31019 | 0.025316 | 0.333778 | 0.1313 | 2.5421 | 0.014374 |
| Q_diazepam | 0.323903 | 0.1303 | 2.48582 | 0.016538 | 0.357598 | 0.131 | 2.72975 | 0.0088919 | 0.32187 | 0.1313 | 2.45141 | 0.018003 |

**ANOVA Summary:**

|  | Admission | | | |  | Hospital | | | |  |  | | Discharge | | | |
| --- | --- | --- | --- | --- | --- | --- | --- | --- | --- | --- | --- | --- | --- | --- | --- | --- |
| Source | **Df** | **Sum**  **squares** | **Mean Squares** | **F** | **Pr(>F)** | **Df** | **Sum**  **squares** | **Mean Squares** | **F** | **Pr(>F)** | **Df** | **Sum**  **squares** | | **Mean Squares** | **F** | **Pr(>F)** |
| Model | 2 | 13.8879 | 6.9439 | 9.2949 | 3.9686E-04 | 2 | 14.5633 | 7.2817 | 9.9382 | 2.5142E-04 | 2 | 14.2718 | | 7.1359 | 9.6575 | 3.0650E-04 |
| Error | 47 | 35.1121 | 0.7471 |  |  | 47 | 34.4367 | 0.7327 |  |  | 47 | 34.7282 | | 0.7389 |  |  |
| Total | 49 | 49 |  |  |  | 49 | 49 |  |  |  | 49 | 49 | |  |  |  |

**Table S7: Prediction of number of Sertraline prescriptions based on number of depressed patients and diazepam prescription.**

**Regression Summary:**

| Regression key values/Time | Admission | Hospital | Discharge |
| --- | --- | --- | --- |
| Regression equation | - | - | -0.0 + (0.355*Q10_2_yes_depress_num) + (0.3255*Q38_9_Diazepam_num) |
| Coefficient of determination  (r-squared) | - | - | 0.3067 |
| Adjusted  r-squared | - | - | 0.2772 |
| Root Mean Square Error (RMSE) | - | - | 0.8243 |
| Mean of Y | - | - | -0.0 |
| Residual standard error | - | - | 0.8502 |
| No. of Observations | - | - | 50 |

**Regression Coefficients:**

| Regression coefficients/  Time | Admission | | | | Hospital | | | | Discharge | | | |
| --- | --- | --- | --- | --- | --- | --- | --- | --- | --- | --- | --- | --- |
| Parameter | **Est** | **Std Err** | **T-val** | **P-val** | **Est** | **Std Err** | **T-val** | **P-val** | **Est** | **Std Err** | **T-val** | **P-val** |
| Intercept | - | - | - | - | - | - | - | - | -1.42872e-10 | 0.1202 | -1.18862e-09 | 1 |
| Q10_depress | - | - | - | - | - | - | - | - | 0.354975 | 0.1283 | 2.76676 | 0.0080702 |
| Q_38_9_diazepam | - | - | - | - | - | - | - | - | 0.325533 | 0.1283 | 2.53728 | 0.014549 |

**ANOVA Summary:**

|  | Admission | | | |  | Hospital | | | |  |  | | Discharge | | | |
| --- | --- | --- | --- | --- | --- | --- | --- | --- | --- | --- | --- | --- | --- | --- | --- | --- |
| Source | **Df** | **Sum**  **squares** | **Mean Squares** | **F** | **Pr(>F)** | **Df** | **Sum**  **squares** | **Mean Squares** | **F** | **Pr(>F)** | **Df** | **Sum**  **squares** | | **Mean Squares** | **F** | **Pr(>F)** |
| Model | - | - | - | - | - | - | - | - | - | - | 2 | 15.0291 | | 7.5145 | 10.3966 | 1.8257E-04 |
| Error | - | - | - |  |  | - | - | - |  |  | 47 | 33.9709 | | 0.7228 |  |  |
| Total | - | - |  |  |  | - | - |  |  |  | 49 | 49 | |  |  |  |

**Table S8: Prediction of number of Citalopram prescriptions based on number of valproate and diazepam prescription.**

**Regression Summary:**

| Regression key values/Time | Admission | Hospital | Discharge |
| --- | --- | --- | --- |
| Regression equation | 0.0 + (0.3821*Q22_4_Valproate_num) + (0.3623*Q24_9_Diazepam_num) | - | - |
| Coefficient of determination  (r-squared) | 0.3167 | - | - |
| Adjusted  r-squared | 0.2876 | - | - |
| Root Mean Square Error (RMSE) | 0.8183 | - | - |
| Mean of Y | 0.0 | - | - |
| Residual standard error | 0.844 | - | - |
| No. of Observations | 50 | - | - |

**Regression Coefficients:**

| Regression coefficients/  Time | Admission | | | | Hospital | | | | Discharge | | | |
| --- | --- | --- | --- | --- | --- | --- | --- | --- | --- | --- | --- | --- |
| Parameter | **Est** | **Std Err** | **T-val** | **P-val** | **Est** | **Std Err** | **T-val** | **P-val** | **Est** | **Std Err** | **T-val** | **P-val** |
| Intercept | 2.04053e-10 | 0.1194 | 1.70899e-09 | 1 | - | - | - | - | - | - | - | - |
| Q_valoprate | 0.38206 | 0.1218 | 3.13678 | 0.0029457 | - | - | - | - | - | - | - | - |
| Q_diazepam | 0.362283 | 0.1218 | 2.97441 | 0.0046224 | - | - | - | - | - | - | - | - |

**ANOVA Summary:**

|  | Admission | | | |  | Hospital | | | |  |  | | Discharge | | | |
| --- | --- | --- | --- | --- | --- | --- | --- | --- | --- | --- | --- | --- | --- | --- | --- | --- |
| Source | **Df** | **Sum**  **squares** | **Mean Squares** | **F** | **Pr(>F)** | **Df** | **Sum**  **squares** | **Mean Squares** | **F** | **Pr(>F)** | **Df** | **Sum**  **squares** | | **Mean Squares** | **F** | **Pr(>F)** |
| Model | 2 | 15.5178 | 7.7589 | 10.8915 | 1.2987E-04 | - | - | - | - | - | - | - | | - | - | - |
| Error | 47 | 33.4822 | 0.7124 |  |  | - | - | - |  |  | - | - | | - |  |  |
| Total | 49 | 49 |  |  |  | - | - |  |  |  | - | - | |  |  |  |

**Table S9: Prediction of number of Citalopram prescriptions based on number of citalopram and diazepam prescription.**

**Regression Summary:**

| Regression key values/Time | Admission | Hospital | Discharge |
| --- | --- | --- | --- |
| Regression equation | - | -0.0 + (0.9266*Q23_4_Citalopram_num) + (0.0916*Q24_9_Diazepam_num) | - |
| Coefficient of determination  (r-squared) | - | 0.9378 | - |
| Adjusted  r-squared | - | 0.9352 | - |
| Root Mean Square Error (RMSE) | - | 0.2469 | - |
| Mean of Y | - | 0.0 | - |
| Residual standard error | - | 0.2548 | - |
| No. of Observations | - | 50 | - |

**Regression Coefficients:**

| Regression coefficients/  Time | Admission | | | | Hospital | | | | Discharge | | | |
| --- | --- | --- | --- | --- | --- | --- | --- | --- | --- | --- | --- | --- |
| Parameter | **Est** | **Std Err** | **T-val** | **P-val** | **Est** | **Std Err** | **T-val** | **P-val** | **Est** | **Std Err** | **T-val** | **P-val** |
| Intercept | - | - | - | - | -7.11931e-11 | 0.036 | -1.97759e-09 | 1 | - | - | - | - |
| Q_citalopram | - | - | - | - | 0.926609 | 0.04 | 23.1652 | 2.3505e-27 | - | - | - | - |
| Q_diazepam | - | - | - | - | 0.0916413 | 0.04 | 2.29103 | 0.026491 | - | - | - | - |

**ANOVA Summary:**

|  | Admission | | | |  | Hospital | | | |  |  | | Discharge | | | |
| --- | --- | --- | --- | --- | --- | --- | --- | --- | --- | --- | --- | --- | --- | --- | --- | --- |
| Source | **Df** | **Sum**  **squares** | **Mean Squares** | **F** | **Pr(>F)** | **Df** | **Sum**  **squares** | **Mean Squares** | **F** | **Pr(>F)** | **Df** | **Sum**  **squares** | | **Mean Squares** | **F** | **Pr(>F)** |
| Model | - | - | - | - | - | 2 | 45.9513 | 22.9756 | 354.1968 | 4.5418E-29 | - | - | | - | - | - |
| Error | - | - | - |  |  | 47 | 3.04874 | 0.0649 |  |  | - | - | | - |  |  |
| Total | - | - |  |  |  | 49 | 49 |  |  |  | - | - | |  |  |  |

**Table S10: Prediction of number of venlafaxine prescriptions based on the number of Frontotemporal and VaD patients.**

**Regression Summary:**

| Regression key values/Time | Admission | Hospital | Discharge |
| --- | --- | --- | --- |
| Regression equation | 0.0 + (0.3904*Q9_dem_subtype_num_Frontotemporal) + (0.3207*Q9_dem_subtype_num_Vascular) | -0.0 + (0.4039*Q9_dem_subtype_num_Frontotemporal) + (0.3303*Q9_dem_subtype_num_Vascular) | -0.0 + (0.4179*Q9_dem_subtype_num_Frontotemporal) + (0.3017*Q9_dem_subtype_num_Vascular) |
| Coefficient of determination  (r-squared) | 0.3184 | 0.3396 | 0.3292 |
| Adjusted  r-squared | 0.2894 | 0.3115 | 0.3007 |
| Root Mean Square Error (RMSE) | 0.8173 | 0.8045 | 0.8108 |
| Mean of Y | 0.0 | -0.0 | 0.0 |
| Residual standard error | 0.843 | 0.8298 | 0.8362 |
| No. of Observations | 50 | 50 | 50 |

**Regression Coefficients:**

| Regression coefficients/  Time | Admission | | | | Hospital | | | | Discharge | | | |
| --- | --- | --- | --- | --- | --- | --- | --- | --- | --- | --- | --- | --- |
| Parameter | **Est** | **Std Err** | **T-val** | **P-val** | **Est** | **Std Err** | **T-val** | **P-val** | **Est** | **Std Err** | **T-val** | **P-val** |
| Intercept | 1.03955e-10 | 0.1192 | 8.72104e-10 | 1 | -2.98021e-10 | 0.1173 | -2.54068e-09 | 1 | -8.22998e-13 | 0.1183 | -6.95687e-12 | 1 |
| Q9_dem_subtype_num_Frontotemporal | 0.39037 | 0.1244 | 3.13802 | 0.0029354 | 0.403923 | 0.1225 | 3.29733 | 0.0018642 | 0.417853 | 0.1235 | 3.38342 | 0.0014519 |
| Q9_dem_subtype_num_Vascular | 0.320702 | 0.1244 | 2.57799 | 0.013133 | 0.330322 | 0.1225 | 2.6965 | 0.0096954 | 0.301673 | 0.1235 | 2.4427 | 0.018391 |

**ANOVA Summary:**

|  | Admission | | | |  | Hospital | | | |  |  | | Discharge | | | |
| --- | --- | --- | --- | --- | --- | --- | --- | --- | --- | --- | --- | --- | --- | --- | --- | --- |
| Source | **Df** | **Sum**  **squares** | **Mean Squares** | **F** | **Pr(>F)** | **Df** | **Sum**  **squares** | **Mean Squares** | **F** | **Pr(>F)** | **Df** | **Sum**  **squares** | | **Mean Squares** | **F** | **Pr(>F)** |
| Model | 2 | 15.6016 | 7.8008 | 10.9777 | 1.2245E-04 | 2 | 16.6395 | 8.3197 | 12.0835 | 5.8312E-05 | 2 | 16.131 | | 8.0655 | 11.533 | 8.4114E-05 |
| Error | 47 | 33.3984 | 0.7106 |  |  | 47 | 32.3605 | 0.6885 |  |  | 47 | 32.869 | | 0.6993 |  |  |
| Total | 49 | 49 |  |  |  | 49 | 49 |  |  |  | 49 | 49 | |  |  |  |

**Table S11: Prediction of number of venlafaxine prescriptions based on the number of citalopram and previous venlafaxine prescriptions.**

**Regression Summary:**

| Regression key values/Time | Admission | Hospital | Discharge |
| --- | --- | --- | --- |
| Regression equation | - | -0.0 + (0.9196*Q23_30_Venlafaxine_num) + (0.1043*Q30_4_Citalopram_num) | - |
| Coefficient of determination  (r-squared) | - | 0.9263 | - |
| Adjusted  r-squared | - | 0.9232 | - |
| Root Mean Square Error (RMSE) | - | 0.2687 | - |
| Mean of Y | - | -0.0 | - |
| Residual standard error | - | 0.2771 | - |
| No. of Observations | - | 50 | - |

**Regression Coefficients:**

| Regression coefficients/  Time | Admission | | | | Hospital | | | | Discharge | | | |
| --- | --- | --- | --- | --- | --- | --- | --- | --- | --- | --- | --- | --- |
| Parameter | **Est** | **Std Err** | **T-val** | **P-val** | **Est** | **Std Err** | **T-val** | **P-val** | **Est** | **Std Err** | **T-val** | **P-val** |
| Intercept | - | - | - | - | -3.91309e-10 | 0.0392 | -9.98238e-09 | 1 | - | - | - | - |
| Q23_30_Venlafaxine_num | - | - | - | - | 0.919614 | 0.0425 | 21.638 | 4.4288e-26 | - | - | - | - |
| Q30_4_Citalopram_num | - | - | - | - | 0.104272 | 0.0425 | 2.45346 | 0.017912 | - | - | - | - |

**ANOVA Summary:**

|  | Admission | | | |  | Hospital | | | |  |  | | Discharge | | | |
| --- | --- | --- | --- | --- | --- | --- | --- | --- | --- | --- | --- | --- | --- | --- | --- | --- |
| Source | **Df** | **Sum**  **squares** | **Mean Squares** | **F** | **Pr(>F)** | **Df** | **Sum**  **squares** | **Mean Squares** | **F** | **Pr(>F)** | **Df** | **Sum**  **squares** | | **Mean Squares** | **F** | **Pr(>F)** |
| Model | - | - | - | - | - | 2 | 45.3904 | 22.6952 | 295.5115 | 2.4024E-27 | - | - | | - | - | - |
| Error | - | - | - |  |  | 47 | 3.60959 | 0.0768 |  |  | - | - | | - |  |  |
| Total | - | - |  |  |  | 49 | 49 |  |  |  | - | - | |  |  |  |

**Table S12: Prediction of number of mirtazapine prescriptions based on the number of frontotemporal patients**

**Regression Summary:**

| Regression key values/Time | Admission | Hospital | Discharge |
| --- | --- | --- | --- |
| Regression equation | 0.0 + (0.4795*Q9_dem_subtype_num_Frontotemporal) | -0.0 + (0.4948*Q9_dem_subtype_num_Frontotemporal) | -0.0 + (0.4993*Q9_dem_subtype_num_Frontotemporal) |
| Coefficient of determination  (r-squared) | 0.2299 | 0.2449 | 0.2493 |
| Adjusted  r-squared | 0.2139 | 0.2292 | 0.2337 |
| Root Mean Square Error (RMSE) | 0.8687 | 0.8602 | 0.8577 |
| Mean of Y | 0.0 | -0.0 | 0.0 |
| Residual standard error | 0.8866 | 0.878 | 0.8754 |
| No. of Observations | 50 | 50 | 50 |

**Regression Coefficients:**

| Regression coefficients/  Time | Admission | | | | Hospital | | | | Discharge | | | |
| --- | --- | --- | --- | --- | --- | --- | --- | --- | --- | --- | --- | --- |
| Parameter | **Est** | **Std Err** | **T-val** | **P-val** | **Est** | **Std Err** | **T-val** | **P-val** | **Est** | **Std Err** | **T-val** | **P-val** |
| Intercept | 3.27778e-12 | 0.1254 | 2.61386e-11 | 1 | -7.91729e-11 | 0.1242 | -6.37463e-10 | 1 | -3.98928e-11 | 0.1238 | -3.22236e-10 | 1 |
| Q9_dem_subtype_num_Frontotemporal | 0.479514 | 0.1267 | 3.78464 | 0.0004276 | 0.49483 | 0.1254 | 3.94601 | 0.0002585 | 0.49933 | 0.1251 | 3.99145 | 0.00022399 |

**ANOVA Summary:**

|  | Admission | | | |  | Hospital | | | |  |  | | Discharge | | | |
| --- | --- | --- | --- | --- | --- | --- | --- | --- | --- | --- | --- | --- | --- | --- | --- | --- |
| Source | **Df** | **Sum**  **squares** | **Mean Squares** | **F** | **Pr(>F)** | **Df** | **Sum**  **squares** | **Mean Squares** | **F** | **Pr(>F)** | **Df** | **Sum**  **squares** | | **Mean Squares** | **F** | **Pr(>F)** |
| Model | 1 | 11.2667 | 11.2667 | 14.3323 | 4.2607E-04 | 1 | 11.998 | 11.998 | 15.5641 | 2.5921E-04 | 1 | 12.2172 | | 12.2172 | 15.9429 | 2.2299E-04 |
| Error | 48 | 37.7333 | 0.7861 |  |  | 48 | 37.002 | 0.7709 |  |  | 48 | 36.7828 | | 0.7663 |  |  |
| Total | 49 | 49 |  |  |  | 49 | 49 |  |  |  | 49 | 49 | |  |  |  |

**Table S13: Prediction of number of lamotrigine prescriptions based on frontotemporal dementia, valproate and dosulepin prescriptions.**

**Regression Summary:**

| Regression key values/Time | Admission | Hospital | Discharge |
| --- | --- | --- | --- |
| Regression equation | 0.0 + (0.3776*Q9_dem_subtype_num_Frontotemporal) + (0.2365*Q22_4_Valproate_num) + (0.433*Q23_6_Dosulepin_num) | - | - |
| Coefficient of determination  (r-squared) | 0.4684 | - | - |
| Adjusted  r-squared | 0.4337 | - | - |
| Root Mean Square Error (RMSE) | 0.7218 | - | - |
| Mean of Y | 0.0 | - | - |
| Residual standard error | 0.7525 | - | - |
| No. of Observations | 50 | - | - |

**Regression Coefficients:**

| Regression coefficients/  Time | Admission | | | | Hospital | | | | Discharge | | | |
| --- | --- | --- | --- | --- | --- | --- | --- | --- | --- | --- | --- | --- |
| Parameter | **Est** | **Std Err** | **T-val** | **P-val** | **Est** | **Std Err** | **T-val** | **P-val** | **Est** | **Std Err** | **T-val** | **P-val** |
| Intercept | 1.32225e-10 | 0.1064 | 1.24272e-09 | 1 | - | - | - | - | - | - | - | - |
| Q9_dem_subtype_num_Frontotemporal | 0.377587 | 0.1127 | 3.35037 | 0.0016195 | - | - | - | - | - | - | - | - |
| Q22_4_Valproate_num | 0.236467 | 0.1139 | 2.07609 | 0.043501 | - | - | - | - | - | - | - | - |
| Q23_6_Dosulepin_num | 0.432975 | 0.1088 | 3.97955 | 0.00024293 | - | - | - | - | - | - | - | - |

**ANOVA Summary:**

|  | Admission | | | |  | Hospital | | | |  |  | | Discharge | | | |
| --- | --- | --- | --- | --- | --- | --- | --- | --- | --- | --- | --- | --- | --- | --- | --- | --- |
| Source | **Df** | **Sum**  **squares** | **Mean Squares** | **F** | **Pr(>F)** | **Df** | **Sum**  **squares** | **Mean Squares** | **F** | **Pr(>F)** | **Df** | **Sum**  **squares** | | **Mean Squares** | **F** | **Pr(>F)** |
| Model | 3 | 22.9507 | 7.6502 | 13.5094 | 1.8808E-06 | - | - | - | - | - | - | - | | - | - | - |
| Error | 46 | 26.0493 | 0.5663 |  |  | - | - | - |  |  | - | - | | - |  |  |
| Total | 49 | 49 |  |  |  | - | - |  |  |  | - | - | |  |  |  |

**Table S14: Prediction of number of lamotrigine prescriptions based on frontotemporal dementia and previous lamotrigine prescriptions.**

**Regression Summary:**

| Regression key values/Time | Admission | Hospital | Discharge |
| --- | --- | --- | --- |
| Regression equation | - | 0.0 + (0.2718*Q9_dem_subtype_num_Frontotemporal) + (0.5839*Q22_2_Lamotrigine_num) | - |
| Coefficient of determination  (r-squared) | - | 0.5568 | - |
| Adjusted  r-squared | - | 0.5379 | - |
| Root Mean Square Error (RMSE) | - | 0.659 | - |
| Mean of Y | - | 0.0 | - |
| Residual standard error | - | 0.6797 | - |
| No. of Observations | - | 50 | - |

**Regression Coefficients:**

| Regression coefficients/  Time | Admission | | | | Hospital | | | | Discharge | | | |
| --- | --- | --- | --- | --- | --- | --- | --- | --- | --- | --- | --- | --- |
| Parameter | **Est** | **Std Err** | **T-val** | **P-val** | **Est** | **Std Err** | **T-val** | **P-val** | **Est** | **Std Err** | **T-val** | **P-val** |
| Intercept | - | - | - | - | 1.29804e-10 | 0.0961 | 1.35072e-09 | 1 | - | - | - | - |
| Q9_dem_subtype_num_Frontotemporal | - | - | - | - | 0.271784 | 0.1086 | 2.50261 | 0.015863 | - | - | - | - |
| Q22_2_Lamotrigine_num | - | - | - | - | 0.58388 | 0.1086 | 5.37643 | 2.3315e-06 | - | - | - | - |

**ANOVA Summary:**

|  | Admission | | | |  | Hospital | | | |  |  | | Discharge | | | |
| --- | --- | --- | --- | --- | --- | --- | --- | --- | --- | --- | --- | --- | --- | --- | --- | --- |
| Source | **Df** | **Sum**  **squares** | **Mean Squares** | **F** | **Pr(>F)** | **Df** | **Sum**  **squares** | **Mean Squares** | **F** | **Pr(>F)** | **Df** | **Sum**  **squares** | | **Mean Squares** | **F** | **Pr(>F)** |
| Model | - | - | - | - | - | 2 | 27.2839 | 13.6419 | 29.5251 | 4.9519E-09 | - | - | | - | - | - |
| Error | - | - | - |  |  | 47 | 21.7161 | 0.462 |  |  | - | - | | - |  |  |
| Total | - | - |  |  |  | 49 | 49 |  |  |  | - | - | |  |  |  |

**Table S15: Prediction of number of lamotrigine prescriptions based on lamotrigine prescriptions during hospitalisation.**

**Regression Summary:**

| Regression key values/Time | Admission | Hospital | Discharge |
| --- | --- | --- | --- |
| Regression equation | - | - | -0.0 + (0.8924*Q29_2_Lamotrigine_num) |
| Coefficient of determination  (r-squared) | - | - | 0.7964 |
| Adjusted  r-squared | - | - | 0.7922 |
| Root Mean Square Error (RMSE) | - | - | 0.4467 |
| Mean of Y | - | - | -0.0 |
| Residual standard error | - | - | 0.456 |
| No. of Observations | - | - | 50 |

**Regression Coefficients:**

| Regression coefficients/  Time | Admission | | | | Hospital | | | | Discharge | | | |
| --- | --- | --- | --- | --- | --- | --- | --- | --- | --- | --- | --- | --- |
| Parameter | **Est** | **Std Err** | **T-val** | **P-val** | **Est** | **Std Err** | **T-val** | **P-val** | **Est** | **Std Err** | **T-val** | **P-val** |
| Intercept | - | - | - | - | - | - | - | - | -5.96325e-10 | 0.0645 | -9.24534e-09 | 1 |
| Q29_2_Lamotrigine_num | - | - | - | - | - | - | - | - | 0.892385 | 0.0651 | 13.7079 | 3.238e-18 |

**ANOVA Summary:**

|  | Admission | | | |  | Hospital | | | |  |  | | Discharge | | | |
| --- | --- | --- | --- | --- | --- | --- | --- | --- | --- | --- | --- | --- | --- | --- | --- | --- |
| Source | **Df** | **Sum**  **squares** | **Mean Squares** | **F** | **Pr(>F)** | **Df** | **Sum**  **squares** | **Mean Squares** | **F** | **Pr(>F)** | **Df** | **Sum**  **squares** | | **Mean Squares** | **F** | **Pr(>F)** |
| Model | - | - | - | - | - | - | - | - | - | - | 1 | 39.0212 | | 39.0212 | 187.7001 | 3.3073E-18 |
| Error | - | - | - |  |  | - | - | - |  |  | 48 | 9.97878 | | 0.2079 |  |  |
| Total | - | - |  |  |  | - | - |  |  |  | 49 | 49 | |  |  |  |

**Table S16: Prediction of number of Quetiapine prescriptions based on number of AD patients**

**Regression Summary:**

| Regression key values/Time | Admission | Hospital | Discharge |
| --- | --- | --- | --- |
| Regression equation | 0.0 + (0.4025*Q9_dem_subtype_num_Alzheimers) | - | - |
| Coefficient of determination  (r-squared) | 0.162 | - | - |
| Adjusted  r-squared | 0.1455 | - | - |
| Root Mean Square Error (RMSE) | 0.9062 | - | - |
| Mean of Y | 0.0 | - | - |
| Residual standard error | 0.9249 | - | - |
| No. of Observations | 50 | - | - |

**Regression Coefficients:**

| Regression coefficients/  Time | Admission | | | | Hospital | | | | Discharge | | | |
| --- | --- | --- | --- | --- | --- | --- | --- | --- | --- | --- | --- | --- |
| Parameter | **Est** | **Std Err** | **T-val** | **P-val** | **Est** | **Std Err** | **T-val** | **P-val** | **Est** | **Std Err** | **T-val** | **P-val** |
| Intercept | 9.22037e-11 | 0.1308 | 7.04921e-10 | 1 | - | - | - | - | - | - | - | - |
| Q9_Alzheimers | 0.402546 | 0.1321 | 3.04729 | 0.0037474 | - | - | - | - | - | - | - | - |

**ANOVA Summary:**

|  | Admission | | | |  | Hospital | | | |  |  | | Discharge | | | |
| --- | --- | --- | --- | --- | --- | --- | --- | --- | --- | --- | --- | --- | --- | --- | --- | --- |
| Source | **Df** | **Sum**  **squares** | **Mean Squares** | **F** | **Pr(>F)** | **Df** | **Sum**  **squares** | **Mean Squares** | **F** | **Pr(>F)** | **Df** | **Sum**  **squares** | | **Mean Squares** | **F** | **Pr(>F)** |
| Model | 1 | 7.94014 | 7.9401 | 9.2822 | 3.7537E-03 | - | - | - | - | - | - | - | | - | - | - |
| Error | 48 | 41.0599 | 0.8554 |  |  | - | - | - |  |  | - | - | | - |  |  |
| Total | 49 | 49 |  |  |  | - | - |  |  |  | - | - | |  |  |  |

**Table S17: Prediction of number of Quetiapine prescriptions on previous quetiapine prescriptions**

**Regression Summary:**

| Regression key values/Time | Admission | Hospital | Discharge |
| --- | --- | --- | --- |
| Regression equation | - | -0.0 + (0.9207*S2A_Quetiapine_num) | - |
| Coefficient of determination  (r-squared) | - | 0.8476 | - |
| Adjusted  r-squared | - | 0.8444 | - |
| Root Mean Square Error (RMSE) | - | 0.3865 | - |
| Mean of Y | - | 0.0 | - |
| Residual standard error | - | 0.3943 | - |
| No. of Observations | - | 50 | - |

**Regression Coefficients:**

| Regression coefficients/  Time | Admission | | | | Hospital | | | | Discharge | | | |
| --- | --- | --- | --- | --- | --- | --- | --- | --- | --- | --- | --- | --- |
| Parameter | **Est** | **Std Err** | **T-val** | **P-val** | **Est** | **Std Err** | **T-val** | **P-val** | **Est** | **Std Err** | **T-val** | **P-val** |
| Intercept | - | - | - | - | -1.52405e-11 | 0.0558 | -2.73128e-10 | 1 | - | - | - | - |
| S2A_Quetiapine_num | - | - | - | - | 0.920676 | 0.0563 | 16.353 | 2.9495e-21 | - | - | - | - |

**ANOVA Summary:**

|  | Admission | | | |  | Hospital | | | |  |  | | Discharge | | | |
| --- | --- | --- | --- | --- | --- | --- | --- | --- | --- | --- | --- | --- | --- | --- | --- | --- |
| Source | **Df** | **Sum**  **squares** | **Mean Squares** | **F** | **Pr(>F)** | **Df** | **Sum**  **squares** | **Mean Squares** | **F** | **Pr(>F)** | **Df** | **Sum**  **squares** | | **Mean Squares** | **F** | **Pr(>F)** |
| Model | - | - | - | - | - | 1 | 41.5345 | 41.5345 | 267.0506 | 3.0343E-21 | - | - | | - | - | - |
| Error | - | - | - |  |  | 48 | 7.46547 | 0.1555 |  |  | - | - | | - |  |  |
| Total | - | - |  |  |  | 49 | 49 |  |  |  | - | - | |  |  |  |

**Table S18: Prediction of number of Quetiapine prescriptions on previous quetiapine prescriptions and AD patients**

**Regression Summary:**

| Regression key values/Time | Admission | Hospital | Discharge |
| --- | --- | --- | --- |
| Regression equation | - | - | -0.0 + (0.0781*Q9_dem_subtype_num_Alzheimers) + (0.5936*S2A_Quetiapine_num) + (0.364*Q28_22_Quetiapine_num) |
| Coefficient of determination  (r-squared) | - | - | 0.95 |
| Adjusted  r-squared | - | - | 0.9467 |
| Root Mean Square Error (RMSE) | - | - | 0.2214 |
| Mean of Y | - | - | 0.0 |
| Residual standard error | - | - | 0.2309 |
| No. of Observations | - | - | 50 |

**Regression Coefficients:**

| Regression coefficients/  Time | Admission | | | | Hospital | | | | Discharge | | | |
| --- | --- | --- | --- | --- | --- | --- | --- | --- | --- | --- | --- | --- |
| Parameter | **Est** | **Std Err** | **T-val** | **P-val** | **Est** | **Std Err** | **T-val** | **P-val** | **Est** | **Std Err** | **T-val** | **P-val** |
| Intercept | - | - | - | - | - | - | - | - | -2.39264e-11 | 0.0326 | -7.3394e-10 | 1 |
| Q9_dem_subtype_num_Alzheimers | - | - | - | - | - | - | - | - | 0.0781236 | 0.0364 | 2.14625 | 0.037161 |
| S2A_Quetiapine_num | - | - | - | - | - | - | - | - | 0.593634 | 0.0846 | 7.01695 | 8.5941e-09 |
| Q28_22_Quetiapine_num | - | - | - | - | - | - | - | - | 0.363957 | 0.0853 | 4.26679 | 9.7867e-05 |

**ANOVA Summary:**

|  | Admission | | | |  | Hospital | | | |  |  | | Discharge | | | |
| --- | --- | --- | --- | --- | --- | --- | --- | --- | --- | --- | --- | --- | --- | --- | --- | --- |
| Source | **Df** | **Sum**  **squares** | **Mean Squares** | **F** | **Pr(>F)** | **Df** | **Sum**  **squares** | **Mean Squares** | **F** | **Pr(>F)** | **Df** | **Sum**  **squares** | | **Mean Squares** | **F** | **Pr(>F)** |
| Model | - | - | - | - | - | - | - | - | - | - | 3 | 46.5486 | | 15.5162 | 291.1561 | 6.4821E-30 |
| Error | - | - | - |  |  | - | - | - |  |  | 46 | 2.45142 | | 0.0533 |  |  |
| Total | - | - |  |  |  | - | - |  |  |  | 49 |  | |  |  |  |
